# The Fraction of Carbon in Soil Organic Matter as a National‐Scale Soil Process Indicator

**DOI:** 10.1111/gcb.70572

**Published:** 2025-10-30

**Authors:** Sabine Reinsch, Inma Lebron, Lis Wollesen de Jonge, Peter L. Weber, Trine Norgaard, Emmanuel Arthur, Lucas Gomes, Charles Pesch, Karyotis Konstantinos, George Zalidis, Lur Epelde, Marija Romic, Davor Romic, Monika Zovko, Marko Reljic, Jaakko Heikkinen, Christopher Feeney, Laura Bentley, Peter Levy, Elena Vanguelova, Panos Panagos, Florian Schneider, Bernhard Ahrens, Jens Leifeld, Gustaf Hugelius, Bridget A. Emmett, Bernhard J. Cosby, Michele Brentegani, Susan Tandy, Amy Thomas, Maud A. J. van Soest, David A. Robinson

**Affiliations:** ^1^ UK Centre for Ecology and Hydrology Bangor UK; ^2^ Department of Agroecology Aarhus University Tjele Denmark; ^3^ Laboratory of Remote Sensing, Spectroscopy, and GIS, Department of Agriculture Aristotle University of Thessaloniki Thessaloniki Greece; ^4^ NEIKER‐Basque Institute for Agricultural Research and Development Derio Spain; ^5^ University of Zagreb Faculty of Agriculture Zagreb Croatia; ^6^ Natural Resources Institute Jokioinen Finland; ^7^ UK Centre for Ecology and Hydrology Penicuik UK; ^8^ Forest Research Farnham UK; ^9^ European Commission Joint Research Centre (JRC) Ispra Varese Italy; ^10^ Thünen Institute of Climate‐Smart Agriculture Braunschweig Germany; ^11^ Max Planck Institute for Biogeochemistry Jena Germany; ^12^ Agroscope Climate and Agriculture Group Zurich Switzerland; ^13^ Department of Physical Geography and Bolin Centre for Climate Research Stockholm Sweden

**Keywords:** decomposition, humification, land use, loss‐on‐ignition, soil health, soil organic carbon, soil organic matter fractions, soil type

## Abstract

Soil organic matter (SOM) is an important component of ecosystem carbon stocks. Generally, SOM found in mineral and organo‐mineral soils can be categorised into two fractions: particulate organic matter (POM) and mineral‐associated‐organic matter (MAOM), both of which contain soil organic carbon (SOC). Understanding the relationship between SOC and SOM fractions provides insight into SOM decomposition and SOC storage potential. Here we show an intriguingly tight relationship between the fraction of SOC in SOM (denoted as $f_{\mathrm{OC}}$), habitat and soil physical properties, as well as SOC stored in POM and MAOM. This opens up new ways to predict spatial variations in the distribution of POC and MAOC using more widely available $f_{\mathrm{OC}}$ data as a covariate. By compiling 14 datasets and 9503 measurements from across Europe and globally we analysed $f_{\mathrm{OC}}$ across mineral and organic soils, which fell between 0.38 and 0.58, consistent with variation in carbon of major plant components. $f_{\mathrm{OC}}$ followed a habitat gradient with lowest median values in Seagrass sediments (0.36 ± 0.09) and Permafrost habitats, followed by croplands (0.47 ± 0.08) and a maximum in semi‐natural habitats (e.g., neutral, acid and calcareous grasslands) (0.56 ± 0.07), with differences between broadleaved (0.50 ± 0.087) and coniferous woodlands (0.53 ± 0.07) which were driven by overall organic matter content. The data show a tight link between vegetation carbon and the contents of SOC and SOM across various habitats, which could be used to inform agricultural soil management, improved land‐use planning (e.g., woodlands), and tracking climate‐related SOC targets.

## Graphical abstract

This research examined the quantity of carbon held in soil organic matter (SOM) across different habitats, using data from nearly 10,000 samples gathered from Europe and other areas. The results revealed a strong relationship between soil categories, types of habitats, and the ability of SOM to store carbon. These insights can help predict soil carbon storage, guide agricultural and land‐use practices, and support climate objectives.

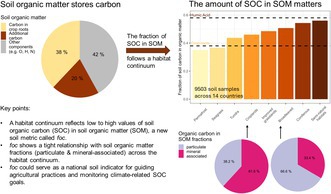

## 1 Introduction

Soil organic carbon (SOC) plays a significant role in various soil functions (Smith et al. 2015), including the mitigation of climate change (Amelung et al. 2020; Lal 2004). The ability of soil to mitigate climate change is rooted in its capacity to ensure that carbon uptake exceeds emissions. By managing soils to consistently increase SOC levels and stocks, it is expected that soils will contribute to the ambitious climate objectives established by the European Union (Panagos et al. 2022) and on a global scale (United Nations 2015). One way of increasing soil carbon stocks is by increasing the organic matter content within soil (Amelung et al. 2020; Poeplau et al. 2015). Organic matter, and consequently soil organic matter (SOM), is inherently heterogeneous (Basile‐Doelsch et al. 2020; Lehmann and Kleber 2015). Organic matter primarily consists of carbon (C), hydrogen (H), nitrogen (N), oxygen (O), and sulphur. It can originate from both plant and animal sources and is processed, or decomposed, by soil biota and microbes, which break it down into progressively smaller molecules. During the decomposition of organic matter, these molecules are used for growth and reproduction, thus changing the stoichiometry of organic matter (Mooshammer et al. 2014; Zechmeister‐Boltenstern et al. 2015). The initial phases of decomposition are frequently examined in studies focusing on the C:N(:Phosphorus) stoichiometry of plant litter (Cools et al. 2014), roots (Silver and Miya 2001), SOM (Zechmeister‐Boltenstern et al. 2015), and the soil microbial community (Cleveland and Liptzin 2007; Mooshammer et al. 2014). Another approach involves analysing the decomposition of organic soils, where the H:C ratio of organic matter is widely used to assess the degree of peatland degradation (Leifeld et al. 2020). The degradation of organic matter results in the formation of biomolecules that can be adsorbed onto mineral surfaces or incorporated into aggregates. Once these biomolecules are immobilised in the soil, they become part of SOM, which has a longer turnover time compared to free biomolecules (Lehmann and Kleber 2015). This concept of SOM formation is referred to as the ‘Soil Continuum Model’ (Lehmann and Kleber 2015) and is recognised as a simplified perspective on the functional complexities associated with SOM and the SOC that persists within (Lehmann et al. 2020).

Consequently, standard metrics like SOM and SOC should provide insights into the balance between the formation and degradation of SOM. Determining the fraction of SOC within SOM, denoted as $f_{\mathrm{OC}}$, we expect that $f_{\mathrm{OC}}$ will follow a decomposition gradient, aligning with the dynamics observed through SOC modelling approaches (Lehmann et al. 2020; Lehmann and Kleber 2015) and the gradual decline of the H:C ratio seen in peatlands (Ahmad and Subawi 2013). For organic soils, the long‐term decomposition of SOM, which results in an increase in carbon density relative to unprocessed SOM, may influence the equilibrium of $f_{\mathrm{OC}}$. In addition to biological processes that impact SOM formation and degradation, $f_{\mathrm{OC}}$ is also influenced by soil type (Batjes 2014; Cools et al. 2014; Silver and Miya 2001; Six et al. 2002), environmental conditions (such as temporal fluctuations in temperature and oxygen) (Lehmann et al. 2020), and global change pressures including land use, land‐use change and global warming (Beillouin et al. 2023).

The literature on soils includes numerous studies in which SOM is used as a predictor for SOC, as it is more straightforward and less expensive to measure (Emmett et al. 2010; Fourqurean et al. 2012; Heikkinen et al. 2021; Maxwell et al. 2023; Pribyl 2010). Pribyl (2010) conducted a review that critiqued the commonly used SOM:SOC ratio of 1.72, asserting that this conversion factor is not universally applicable and serves merely as an empirical upper limit for the correlation between SOM and SOC. A similar viewpoint was expressed by Klingenfuß et al. (2014), who reviewed SOM:SOC ratios employed for peatland soil substrates and advocated for the adoption of conversion factors specific to peat types instead of a single conversion factor for peatland soils. Beyond the debate regarding the application of SOM:SOC ratios, could this established relationship serve as a powerful (and cost‐effective) indicator of soil processes?

**FIGURE 1:**
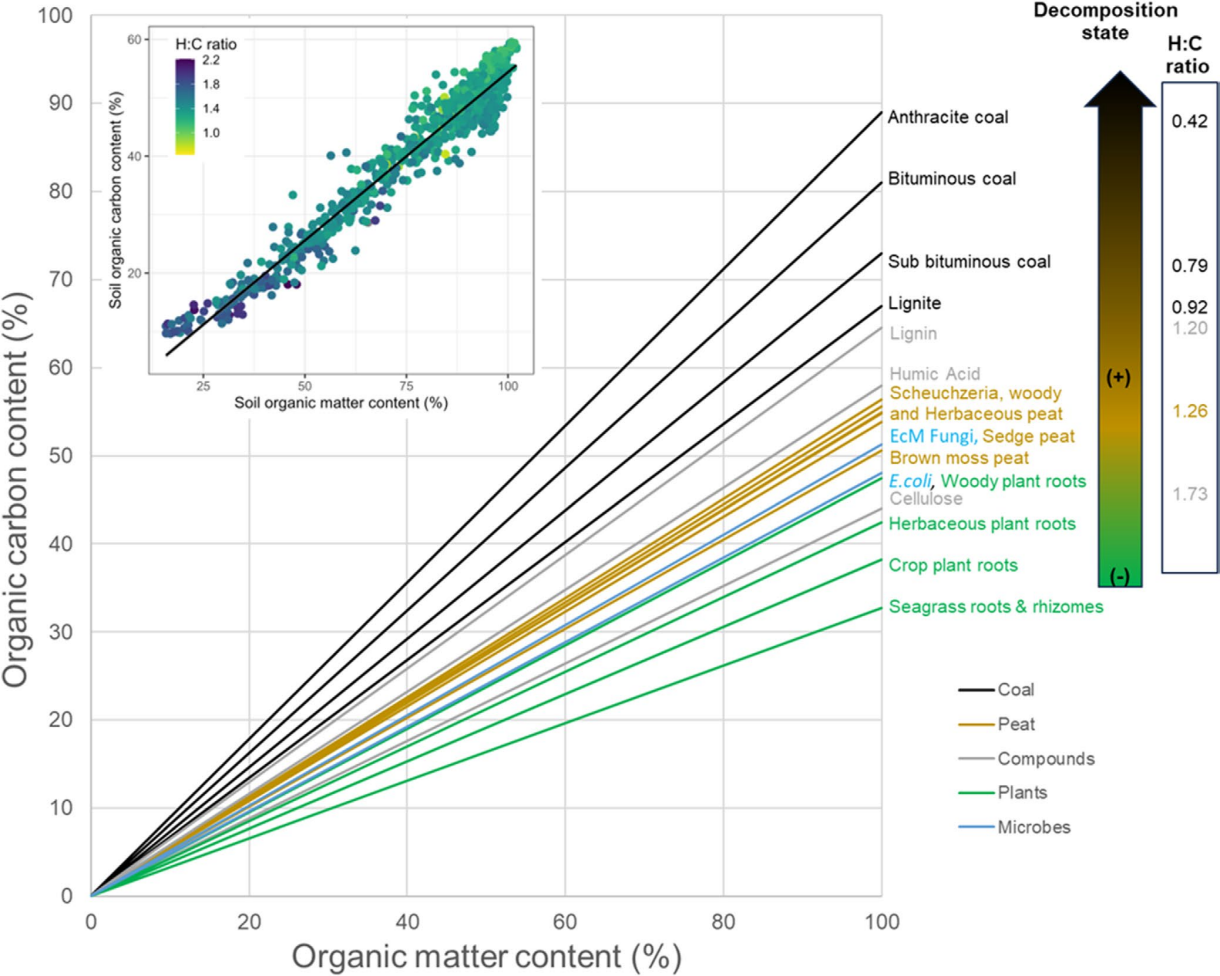
Overview of the relationship between the organic matter and organic carbon contents. The lines illustrate the relationships between organic matter and organic carbon for unprocessed organic matter such as roots (green), microorganisms (blue), cellulose and lignin (grey), which are common components of plants, (partly) decomposed organic matter (brown), and coal (black). The arrow illustrates the variation in H:C ratio of organic matter from the highest to lowest as decomposition progresses. EcM refers to ectomycorrhizal fungi (necromass of *Meliniomyces bicolor*). Lines for crop roots (38% C) and humic acid (58% C) serve as reference carbon contents per unit of organic matter in subsequent figures. The figure insert presents the H:C ratio for peatland soils from Leifeld et al. (2020) as a function of soil organic matter and soil organic carbon contents. The points follow a linear relationship with a slope of 0.54 (*R* ^2^ = 0.9957, *p* < 0.001).

A synthesis of information on the carbon contents of organic matter that may enter the SOC pool is presented in Figure 1; it suggests how the relationship between organic carbon and organic matter is expected to change as decomposition progresses. According to the meta‐analysis conducted by Ma et al. (2018), the carbon contents in roots are lowest in crops (~38%), followed by herbaceous + crop roots (~42%) and roots of woody species (~47%). The majority of incoming organic matter is processed by the soil microbial community, with carbon contents ranging from 47% to 51% for *Escherichia coli* (Heldal et al. 1985) and ectomycorrhizal fungi (Fernandez and Kennedy 2018), respectively. Peats have higher carbon contents relative to their organic matter content, positioned between coal and fresh organic matter (Figure 1).

According to the ‘Soil Continuum Model’ (Basile‐Doelsch et al. 2020; Lehmann and Kleber 2015), the $f_{\mathrm{OC}}$ is expected to range from 0.38 to 0.58, which reflects the carbon contents in organic matter derived from plant input such as crop roots (Ma et al. 2018) and the biomolecule humic acid (Pribyl 2010) that is produced during decomposition processes. Hence, it is expected that biogeochemically ‘unprocessed’ organic matter integrated into SOM will exhibit a carbon‐organic matter relationship with a slope nearer to 0.38, while ‘processed’ organic matter is likely to show a relationship with a slope closer to that of the soil microbial community (~0.5) or higher, due to the degradation of organic matter. Moreover, Figure 1 illustrates representative values for the H:C ratios of cellulose, lignin, peats and coals, emphasising the decrease in the H:C ratio as carbon content increases with decomposition. Additionally, it indicates that carbon accumulation in (Swiss) peatlands is a function of SOM degradation, as evidenced by lower H:C ratios at higher SOM levels.

Identifying soils that have the capacity to sequester additional SOC is a vital step because of the possible saturation limits of SOC (Emmett et al. 2023; Lugato et al. 2021; Six et al. 2002). Particulate organic matter (POM) is created through the aggregation of structural residuals, while mineral‐associated organic matter (MAOM) originates from dissolved organic material or is chemically altered by soil microorganisms (Cotrufo et al. 2015; Lavallee et al. 2020; Rocci et al. 2024). MAOM is protected against decomposition due to its association with soil minerals, unlike POM, which lacks this protection. These two fractions of SOM are believed to create a continuum within mineral and organo‐mineral soils (Cotrufo et al. 2019; Lavallee et al. 2020). As conceptually illustrated by Lavallee et al. (2020), POM primarily comes from plant litter and roots, with average C:N ratios between 10 and 40, with mean residence times spanning from years to decades. In contrast, the formation of MAOM is dependent on the availability of mineral surfaces and low‐molecular‐weight substances derived from microorganisms and plants. MAOM has a narrower C:N ratio range (8–13) compared to POM, and generally has longer mean residence times, potentially lasting for centuries. When occluded in soil aggregates, the residence time of POM can be comparable to that of MAOM due to its limited accessibility for microbial processing (Schmidt et al. 2011). Additional protection of MAOM from decomposition may occur when it is confined within small aggregates (Chi et al. 2022; Even and Francesca Cotrufo 2024; Lavallee et al. 2020), as this reduces physical contact with decomposers. From a management perspective, MAOM is targeted for carbon storage (MAOC) in croplands and grasslands (Lugato et al. 2021), while carbon stored in POM (POC) is managed in wetlands and peatlands. However, the assessment of POC and MAOC is costly and labour‐intensive, prompting the search for an appropriate surrogate to estimate and predict carbon stored in SOM fractions over space and time.

The aim of this paper is to investigate the fraction of carbon in SOM, denoted as $f_{\mathrm{OC}}$, as an indicator of SOM decomposition across European habitats. Our composite dataset encompasses soils from different European regions, extending from the northernmost part in Greenland to Spain (Figure S1; Table S1). It includes Arctic soils and sediments found in Seagrass habitats characterised by low rates of organic matter decomposition, as well as Peatlands with highly decomposed organic matter. We proposed that by consolidating multiple datasets (*n* = 14) across habitats on a continental scale and beyond, we can derive integrated insight on SOM decomposition from the fraction of carbon contained in SOM. Additionally, by using a subset of the UKCEH Countryside Survey data (Reynolds et al. 2013), we obtained data on $f_{\mathrm{OC}}$ and associated SOM fractions (*n* = 92), which allowed us to establish the relationship between POC and MAOC fractions with $f_{\mathrm{OC}}$, serving as a potential proxy for predicting POC and MAOC across different habitats.

## 2 Materials and Methods

### 2.1 Datasets Across Habitats

The Countryside Survey's topsoil (0–15 cm) dataset has reliably demonstrated a relationship of ~0.55 between SOC and SOM over time (Emmett et al. 2010). Building on this finding, we investigated the most current dataset from the UKCEH Countryside Survey (Bentley et al. 2024; Bentley, Reinsch, Alison, Andrews, et al. 2023; Bentley, Reinsch, Alison, Brentegani, et al. 2023; Bentley, Reinsch, Brentegani, Chetiu, et al. 2023; Reinsch, Bentley, et al. 2023) along with the recent Welsh national topsoil dataset (Reinsch, Bentley, et al. 2025) to evaluate the consistency of this relationship across different habitats. To extend our search for datasets in the UK and beyond, we conducted a selective review of the literature for publications concerning (large‐scale) datasets that included SOC and SOM measurements from the same soil samples. As a result of this investigation, we identified datasets focused on croplands (Bentley, Reinsch, Alison, Andrews, et al. 2023; Bentley, Reinsch, Alison, Brentegani, et al. 2023; Heikkinen et al. 2022; Reinsch 2025; Reinsch, Bentley, et al. 2023), peatlands [bog (Leifeld et al. 2020; Toberman et al. 2016), fen, marsh swamps (Palmtag, Obu, Kuhry, Siewert, et al. 2022)], woodlands (Mitchell et al. 2020; Palmtag, Obu, Kuhry, Richter, et al. 2022; Reinsch 2025; Reinsch, Lebron, et al. 2023), grasslands (Reinsch 2025; Reinsch, Lebron, et al. 2023; Weber et al. 2023), the Permafrost region [Yedoma sediment, Tundra, Barren (Palmtag, Obu, Kuhry, Richter, et al. 2022)] and global seagrass habitats (Fourqurean 2012) suitable for our analysis.

All datasets underwent the same data quality checks: SOM values were restricted to a maximum of 100%. Habitat data were essential for this analysis, and all depth data were included provided that the profile showed a continuum of carbon and SOM. There are 9503 data points available, of which 62% are topsoil samples (to a depth of 0.20 m) and 38% are subsoil samples with a maximum depth of 5.65 m (Reinsch, Weber, et al. 2025).

SOM is routinely measured using the loss‐on‐ignition (LOI) method (Lebron et al. 2024), and for Peatlands, it is calculated as the sum of its elements (Leifeld et al. 2020). In contrast, SOC is commonly measured using dry combustion (Lebron et al. 2024). The SOM content is calculated as the difference between the mass of soil samples that were dried at room temperature and the mass observed after combustion up to 375°C, 450°C, or 550°C depending on the employed method (Figure S7). Datasets that used any of these methods for determining SOM were included in the analysis.

The SOC content of fine earth, which was passed through a 2 mm sieve, is determined through elemental analysis of dry soil. The mass of carbon released during the combustion of a sample indicates the mass of carbon produced in the form of CO_2_. For soils that lack inorganic carbon $\left(C_{\text{inorg}}\right)$, SOC corresponds to the total carbon mass. In cases where soils contain $C_{\text{inorg}}$, it must be removed before conducting elemental analysis. Alternatively, $C_{\text{inorg}}$ can be measured by thermographic analysis within the temperature range of 650°C–1000°C (Lebron et al. 2024) and subsequently subtracted from total carbon values. It is crucial to ensure that $C_{\text{inorg}}$ is completely removed or accounted for; otherwise, SOC values may be inflated (see also Figure S10). Methods used to determine total carbon, inorganic carbon, SOC and SOM are compiled in Table S1. We define the fraction of SOC within SOM as:

$$f_{\mathrm{OC}}=\frac{f_{\mathrm{SOC}}}{f_{\mathrm{SOM}}}$$

### 2.2 Data and Data Quality

Data regarding total carbon (%dw), CaCO_3_ (%dw), SOC (%dw), SOM (%dw), and habitat were gathered from all datasets. In cases where total carbon was measured on untreated samples, CaCO_3_‐C was subtracted to calculate SOC. When soils underwent pre‐treated to eliminate carbonates, total carbon was considered as SOC. It was assumed that Peat soils did not contain carbonates and were treated as SOC. The data underwent quality checks using $f_{\mathrm{OC}}$, ensuring that fractions did not fall below 0.27 or exceed 1, except for Seagrass habitats, acknowledging measurement uncertainties and variability of carbon inputs as illustrated in Figure 1.

### 2.3 Comparison of Methods for Measuring SOM

A methodological validation was conducted by Emmett et al. (2010), in which 40 soil samples were selected from a range of SOM. The LOI method, a widely used technique for measuring SOM, was applied to 10 g of soil at 375°C and 1 g of soil at 550°C from the same soil sample. The results showed a strong correlation between the two methods across the LOI gradient (*R* ^2^ = 0.9982), but less SOM was detected at the lower temperature. Additionally, it was observed that the discrepancies in detected SOM were more pronounced at lower organic matter contents (< 20%). Following this observation, 1104 soil samples from the 1998 Countryside Survey soils were re‐analysed for LOI at 375°C, providing a substantial dataset for comparison of LOI at 375°C and 550°C (Figures S5 and S6).

### 2.4 Databases

Fourqurean et al. (2012) compiled a database on seagrass data points that includes original data on SOC and SOM found in sediments (Fourqurean 2012). Out of 3640 data points, 1384 contained SOC and SOM measurements that met the previously stated criteria. Ma et al. (2018) conducted a meta‐analysis examining the carbon content in plants and their respective organs on a global scale. The values from the database were used to illustrate the carbon contents in roots and leaves for both herbaceous and woody plant species (Figure S2). The database did not include seagrass vegetation; therefore, we gathered data for leaves, rhizomes, and roots from the literature (Holmer et al. 2004; Jiang et al. 2019; Luo et al. 2024) to complement Figure S2.

### 2.5 SOM Density Fractionation

SOM density fractionation was conducted in 2022 on 92 archived soil samples (sieved to 2 mm and air dried) collected in 2019 and 2020 (Lebron et al. 2025). A modified and simplified fractionation method to the one described in Reinsch et al. (2024) was used. In short, SOM fractionation was carried out using sodium polytungstate (SPT), with 990 g of SPT dissolved in 810 mL of deionised water to achieve a density of 1.8 g cm^−3^. A subsample of 2.5 g of archived soil was density separated to obtain the light‐free fraction (LF). The remaining pellet was re‐suspended with 1.8 g cm^−3^ SPT and subjected to ultrasonic sonication to disintegrate the soil aggregates. The supernatant containing the occluded carbon fraction was discarded, while the residual material was associated with the MAOM. Both fractions, as well as a bulk soil sample, were analysed for carbon content through combustion as described above. The SOM content was determined using the TGA as described in Lebron et al. (2024).

The occluded light fraction (OF) was obtained through mass balance calculations from total soil, LF, and MAOM. The POM fraction was determined by adding LF and OF together. POM‐C and MAOM‐C, which are referred to as POC and MAOC, respectively, were calculated using the measured carbon concentrations along with mass balance and are expressed as fractions (g fraction per g soil) of SOM. Total carbon and MAOC were corrected for calcite‐C content. LF, MAOM, total soil, and SOC (which is the total carbon minus calcite‐C) were corrected for hygroscopic water content. A detailed description is available in Lebron et al. (2025) and the Supporting Information.

### 2.6 Statistical Analysis

The statistical analysis was conducted to determine whether habitat significantly effected $f_{\mathrm{OC}}$, and if it does, to identify which habitats align with or diverge from the overall slope of the dataset, which is 0.54. The statistical analysis was performed using R version 4.4.1 (R Core Team 2024). A linear mixed effects model (lmer) from the ‘lme4’ package was fitted to $f_{\mathrm{OC}}$ as a function of habitat and an interaction between SOC content and soil type (mineral, organic, permafrost, sediment), using data from *n* = 8936 (*n* = 9503 when TC is included) data points. The soil profile was incorporated as a random effect. The method for measuring SOM and Dataset ID did not increase (but rather decreased) the explanatory power and were therefore excluded from the final model. For additional information, refer to the Supporting Information.

The model assumes a normal distribution of errors which was confirmed through visual inspection (Figure S11). We conducted a visual examination of the data (Figures S11–S14), which identified a relationship between $f_{\mathrm{OC}}$ and SOC (as well as SOM) content by soil category. The final model is a linear hierarchical mixed‐effect model structured as follows:

$$\text{lmer}\left(f_{\mathrm{OC}}\sim \text{Soil category}:\mathrm{SOC}+\text{Habitat}+\left(1|\text{Profile}\right)\right)$$

The profile describes the soil profile from which the measurement is derived. An ANOVA (Type III) was used to evaluate the significance of habitat and soil category:SOC (refer to Table S4). The function *ggpredicts* from the package *ggeffects* was used to predict $f_{\mathrm{OC}}$ based on habitat and soil category. The impact of the soil category on $f_{\mathrm{OC}}$ was established at the observed median $f_{\mathrm{OC}}$ for each category by modifying the *condition* statement within the *ggpredict* function. The condition statements were 0.037, 0.47, 0.061 and 0.021 for mineral, organic, permafrost and sediment categories, respectively. The predicted $f_{\mathrm{OC}}$ values for each habitat were extracted for relevant soil categories only (see Table S5). The slopes reported in the main text correspond to ±1 standard deviation (SD).

The correlation between $f_{\mathrm{OC}}$ and $f_{\mathrm{POC}}$ (Figure 6) was evaluated using the cor.test() function in R, applying a 95% confidence interval. This analysis was performed on the raw data points (plotted in grey) across all habitats.

## 3 Results

Initially, we explored the available carbon and SOM data (*n* = 9503) in relation to habitat and a designated soil category. There was a lack of consistent information regarding soil type or texture, with only 6.3% of data containing associated soil texture information. Consequently, we hierarchically assigned four soil categories based on the limited information available. The reasoning was as follows: sediments were designated for Seagrass habitats and Yedoma sediments. If the data did not fall into this category, then the permafrost category was assigned to data originating from permafrost regions. The remaining data were classed into mineral and organic soils according to their SOM content (< 20% and > 20%, respectively).

**FIGURE 2:**
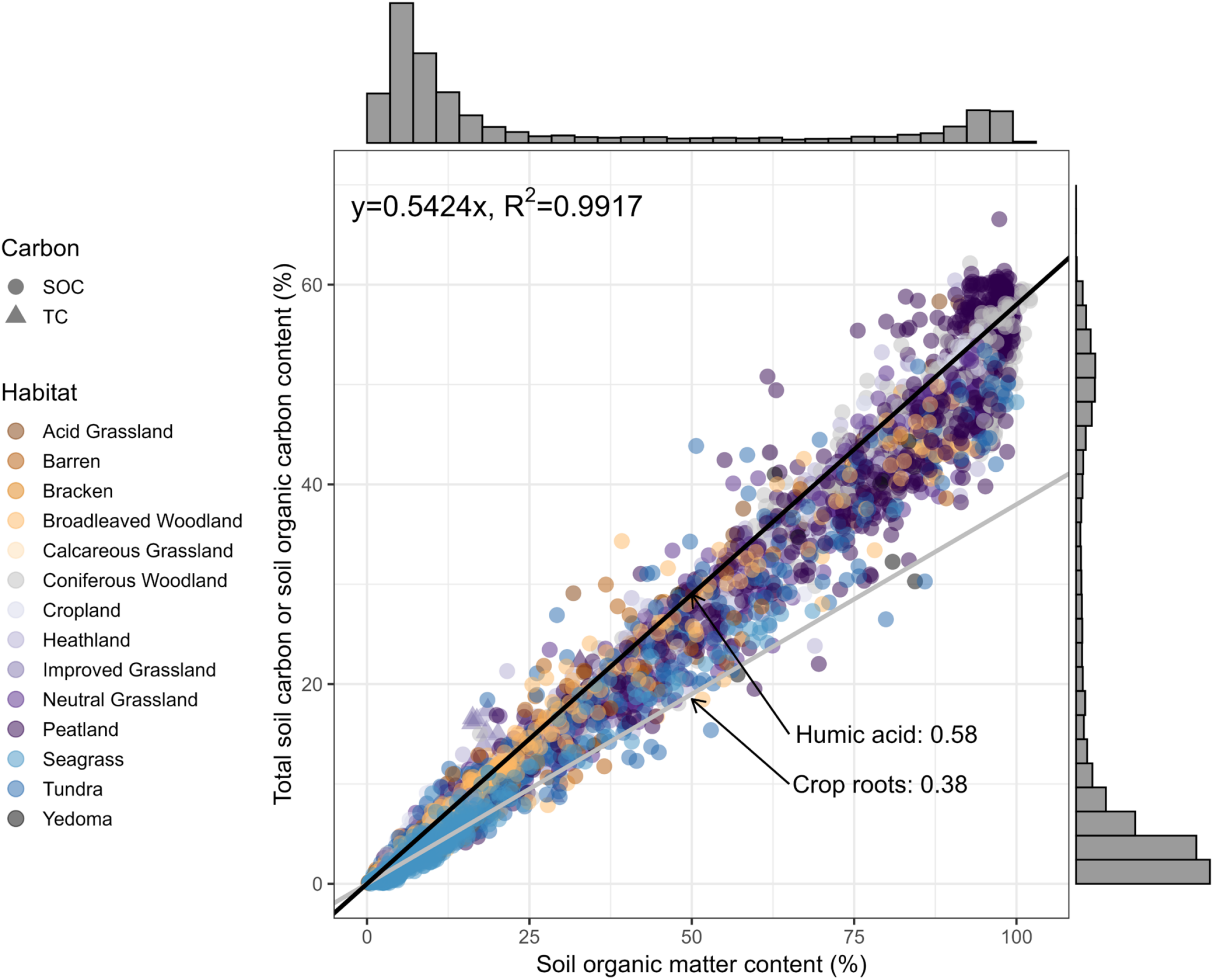
Data for measurements of total soil carbon (represented by triangles, *n* = 567, of which 495 being topsoil samples and 72 being subsoil samples) and soil organic carbon (represented by circles, *n* = 8936, with 5415 being topsoil samples and 3521 being subsoil samples), as well as soil organic matter content across various habitats. The data demonstrate a linear relationship of 0.54. The grey line has a slope of 38%, which corresponds to the average carbon content found in crop roots, while the black line has a slope of 58%, corresponding to the average carbon content of humic acid, as illustrated in Figure 1.

Soil carbon measured across European and Northern permafrost regions (refer to Table S1; Figure S1) and various habitats follows a linear relationship with SOM (Figure 2). The data points fall roughly between slopes of 0.38 and 0.58, with an average slope of 0.54 ± 0.001 (*F* = 1.13e^6^, df = 9502, *p* < 0.001). Habitat significantly affected $f_{\mathrm{OC}}$ (*F* = 70, df = 13, *p* < 0.001) as did the interaction between soil category and SOC (*F* = 528, df = 4, *p* < 0.001). Overall, the fixed effects of habitat and the positive correlation between $f_{\mathrm{OC}}$ and SOC by soil category explained 57% of the variability in the $f_{\mathrm{OC}}$ data. Additionally, the random effect (soil profile) explained another 17% of the variability (refer to Table S4).

**FIGURE 3:**
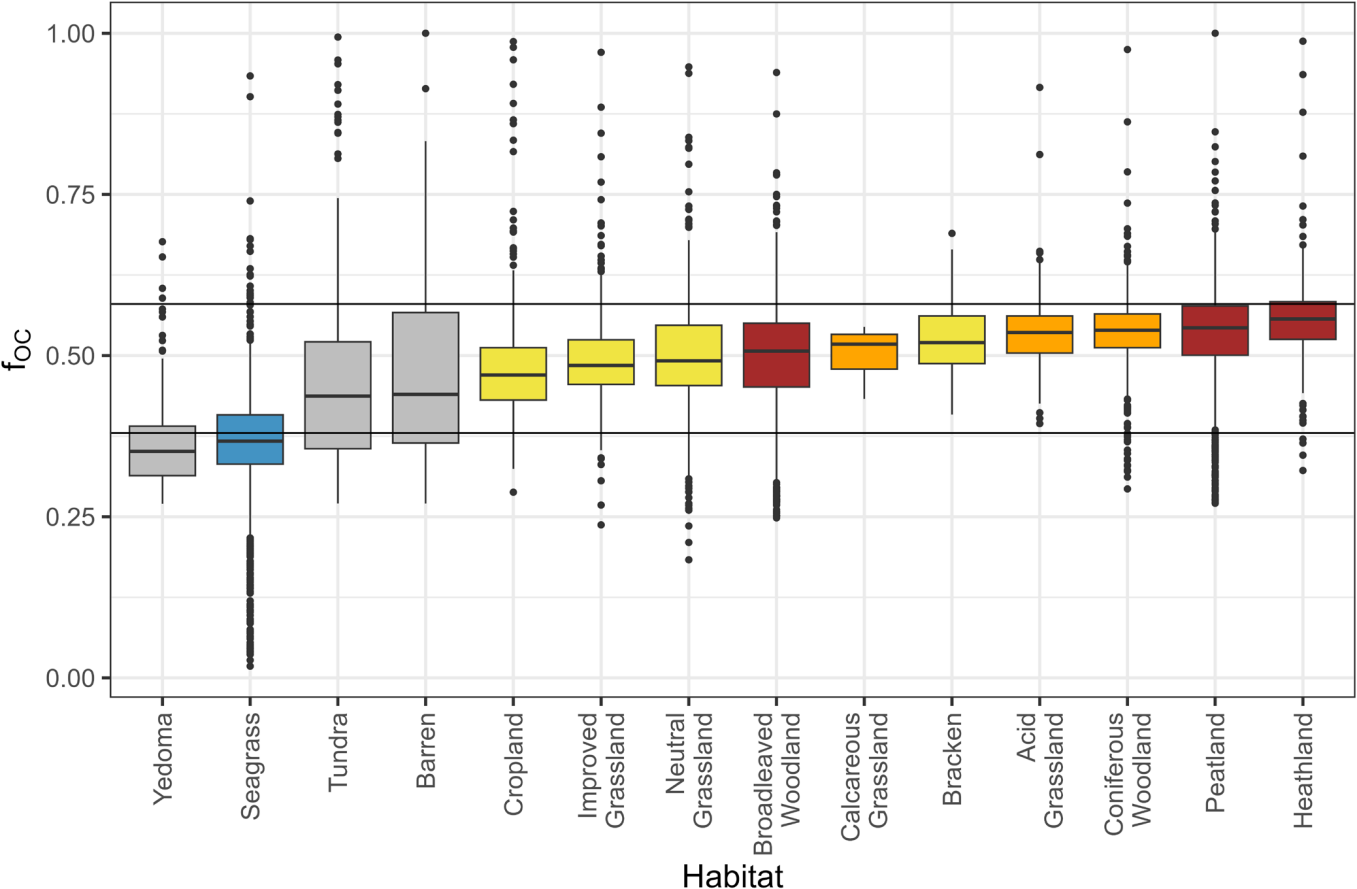
The fraction of organic carbon in soil organic matter $\left(f_{\mathrm{OC}}\right)$ across various habitats. Blue: Seagrass, and grey: Yedoma sediment, Tundra and Barren of the permafrost region where distinct time scales for soil processes are presumed due to the influence of water column or permafrost, respectively; yellow: Herbaceous plants or habitats, brown: Woody plants or woodlands, orange: Semi‐natural habitats sitting between herbaceous and woody vegetation. The horizontal lines reflect the average carbon contents of crop roots (38%, $f_{\mathrm{OC}}=0.38$) and humic acid (58%, $f_{\mathrm{OC}}=0.58$) consistent with Figure 1. Summary statistics relevant to this figure can be found in Table S2.

Dividing the dataset according to habitat, the gradient of $f_{\mathrm{OC}}$ (Figure 3; Table S2) shows that Yedoma sediment has the lowest median $f_{\mathrm{OC}}$ at 0.35, followed by Seagrass sediments at 0.37, and soils from the permafrost region with Tundra at 0.43 and Barren at 0.44. These four habitats show $f_{\mathrm{OC}}$ values that are significantly different from 0.54 (Table S4). Progressing along the $f_{\mathrm{OC}}$ gradient, cropland soils have a median $f_{\mathrm{OC}}$ at 0.46, followed by soils from other non‐woody habitats ranging between 0.48 and 0.49, and broadleaved woodland at 0.51. Semi‐natural habitats present transitional median values for $f_{\mathrm{OC}}$, which are followed by the median $f_{\mathrm{OC}}$ observed in woody vegetation other than broadleaved, specifically coniferous woodland at 0.54 and heathland at 0.56.

The lowest concentration of SOC (as detailed in Table S2) was observed in cropland soils (mean = 0.26 g kg^−1^, range = 4.10 g kg^−1^). This was closely followed by Seagrass sediments (0.30 g kg^−1^, range = 4.82 g kg^−1^). At the opposite end of the spectrum, Peatland soils showed the highest SOC concentration (3.97 g kg^−1^, range = 6.64 g kg^−1^), followed by heathland SOC (2.96 g kg^−1^, range = 5.90 g kg^−1^).

**FIGURE 4:**
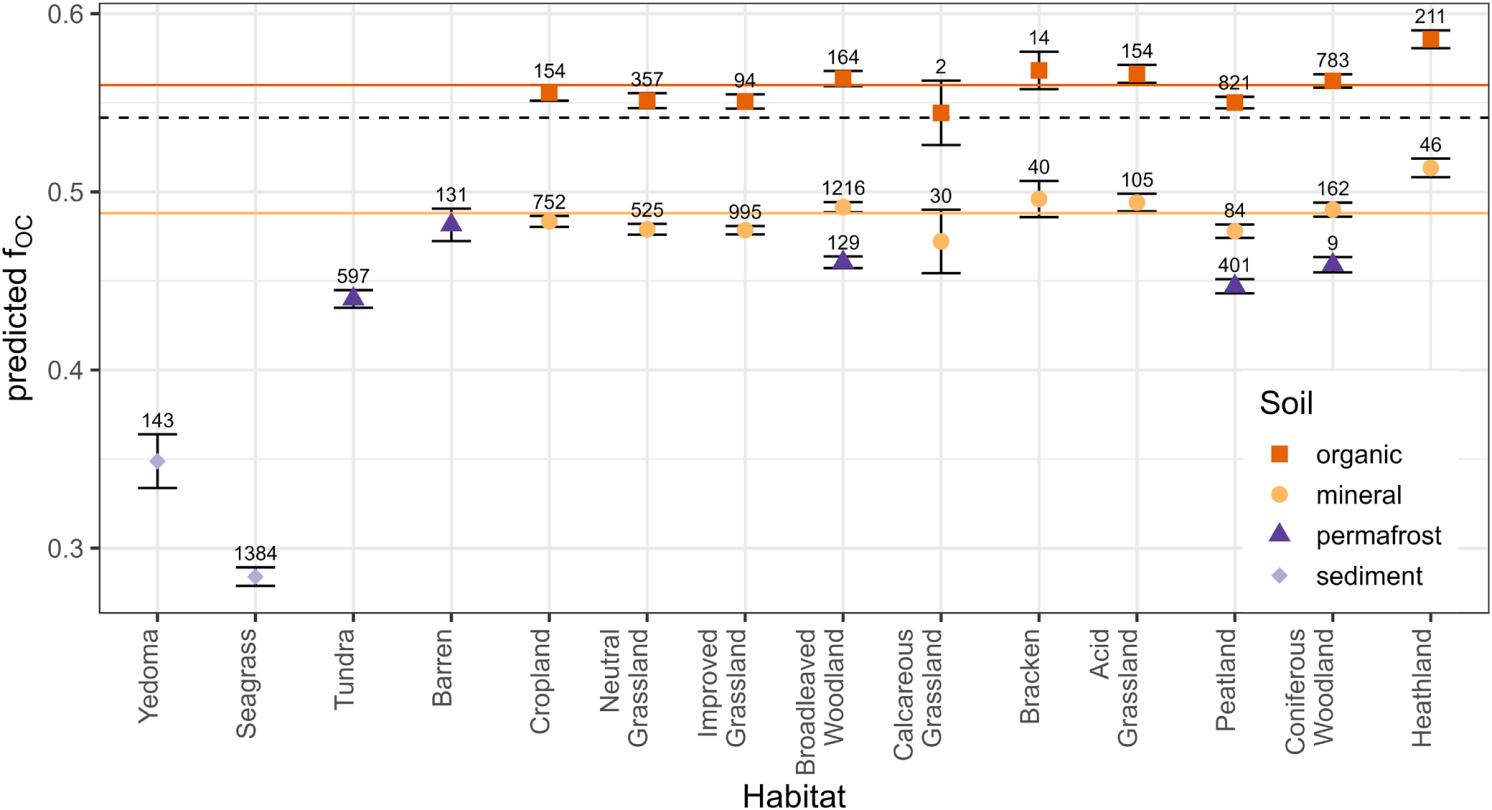
The predicted fraction of organic carbon in soil organic matter $\left(f_{\mathrm{OC}}\right)$ varies across habitats and soil, with mineral soil defined by SOM contents below 20% and organic soils defined contents above that threshold. The data on sediments combine data from Yedoma sediments and Seagrass habitats. Only the observed combinations of soil and habitat combinations are represented in the figure. The numbers of observations are indicated above each error bar. All data from permafrost regions are included in the permafrost category. The dashed horizontal line indicates a slope of 0.54, aligning with Figure 2, while the orange and yellow lines represent the average predicted $f_{\mathrm{OC}}$ values for the organic (0.56) and mineral (0.49) soil categories.

The relationship between SOC and $f_{\mathrm{OC}}$ was found to be positive across all soil categories (Figure S13; Table S4). Soil categories determine the overall level of $f_{\mathrm{OC}}$, with habitat influencing SOC contents (Figure 4). Organic soils demonstrate the highest predicted $f_{\mathrm{OC}}$, averaging 0.56 ± 0.01 (SD) compared to mineral soils (0.49 ± 0.01 SD). Permafrost soils show a similar average predicted $f_{\mathrm{OC}}$ (0.46 ± 0.02 SD) to mineral soils, while sediments present the lowest average predicted $f_{\mathrm{OC}}$ values with at 0.32 ± 0.05 (SD). Overall, the $f_{\mathrm{OC}}$ gradient illustrated in Figure 3 indicates that mineral soils typically possess lower $f_{\mathrm{OC}}$ values, whereas organic soils show higher $f_{\mathrm{OC}}$ values. This trend may serve as a useful indicator of SOC storage potential at both national and continental scales.

**FIGURE 5:**
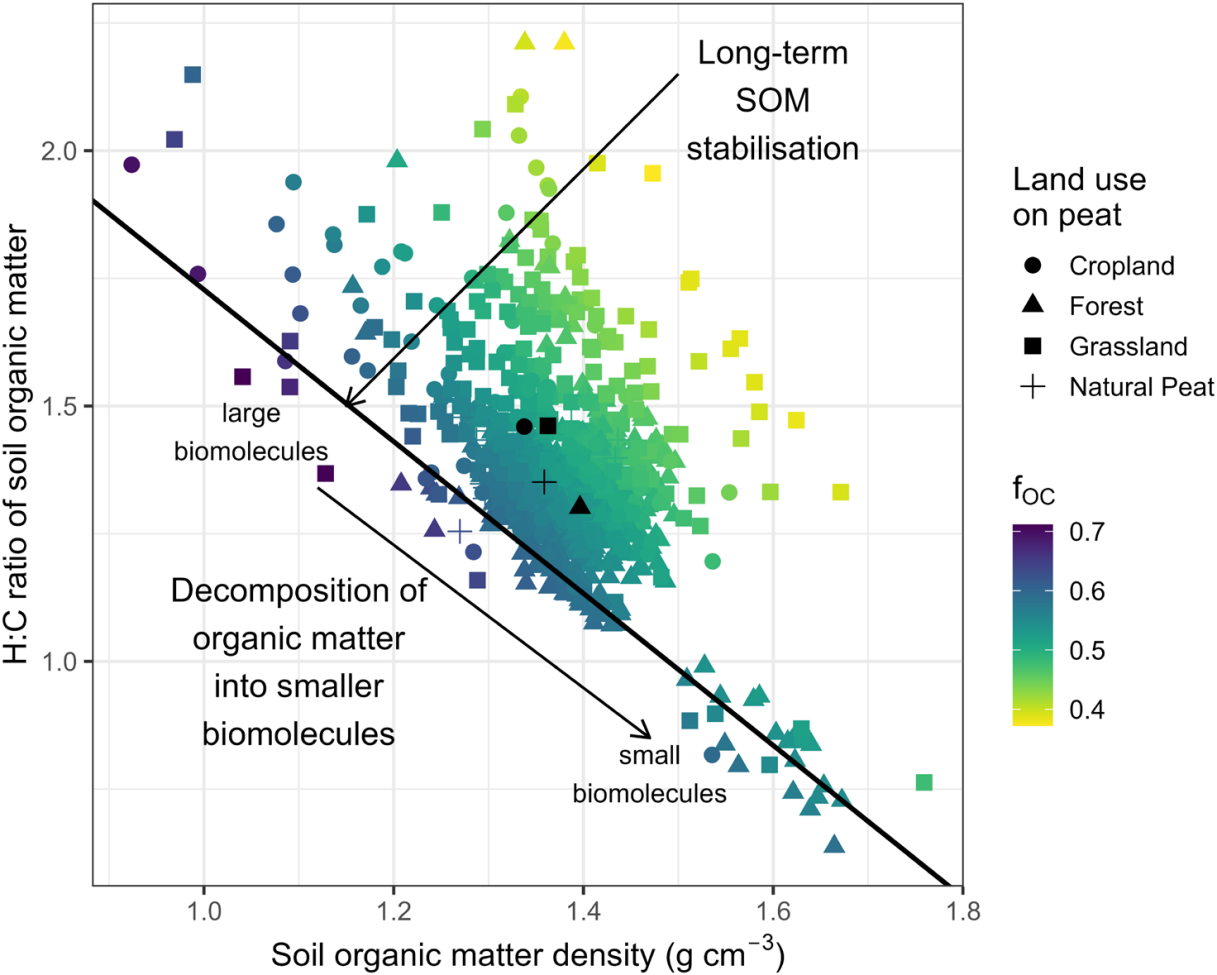
The density of soil organic matter (SOM, g cm^−3^) is plotted against the H:C ratio of peatland SOM, serving as an indicator of SOM decomposition, using data from Leifeld et al. (2020). The colour scale for $f_{\mathrm{OC}}$ ranges from 0.7 to 0.4, corresponding to high and low carbon content in SOM, respectively. Black shapes represent the average SOM densities and H:C ratios for each type of land use on peat. The solid black line indicates the lower 5% percentile of the data, marking the lower boundary and thus the equilibrium between organic matter decomposition and SOM density. The two arrows represent the direction of SOM decomposition and long‐term stabilisation of SOM. According to the consolidated view of Lehmann and Kleber (2015), the decomposition of organic matter occurs along the lower boundary, while the long‐term stabilisation of SOM may account for the gradient of $f_{\mathrm{OC}}$ that runs parallel to that lower boundary. The calculation of SOM density was performed by applying the conversion factor from Kuwata et al. (2012) to the Swiss peatland dataset (Leifeld et al. 2020) (Figure S3).

The unique dataset on peatlands from Leifeld et al. (2020) provides an opportunity to investigate the impact of land use (or habitat) on $f_{\mathrm{OC}}$ for organic soils. The differentiation of habitats on peat was evident in the H:C ratios, which ranged from 0.65 to 2.1. The H:C ratios for forest on peat (1.30 ± 0.14) and natural peat (1.35 ± 0.06) are lower than those for croplands (1.46 ± 0.21) and grasslands (1.46 ± 0.20) on Peat, indicating that the latter contains a smaller amount of decomposed organic matter. H:C ratios were observed to decline as SOM density increased (Figure 5; Figure S3). The density of SOM increases in relation to the decomposition of organic matter from peat under cropland (1.34 ± 0.10 g cm^−3^), grasslands (1.36 ± 0.09 g cm^−3^), natural peats (1.36 ± 0.04 g cm^−3^), to forests (1.40 ± 0.06 g cm^−3^).

**FIGURE 6:**
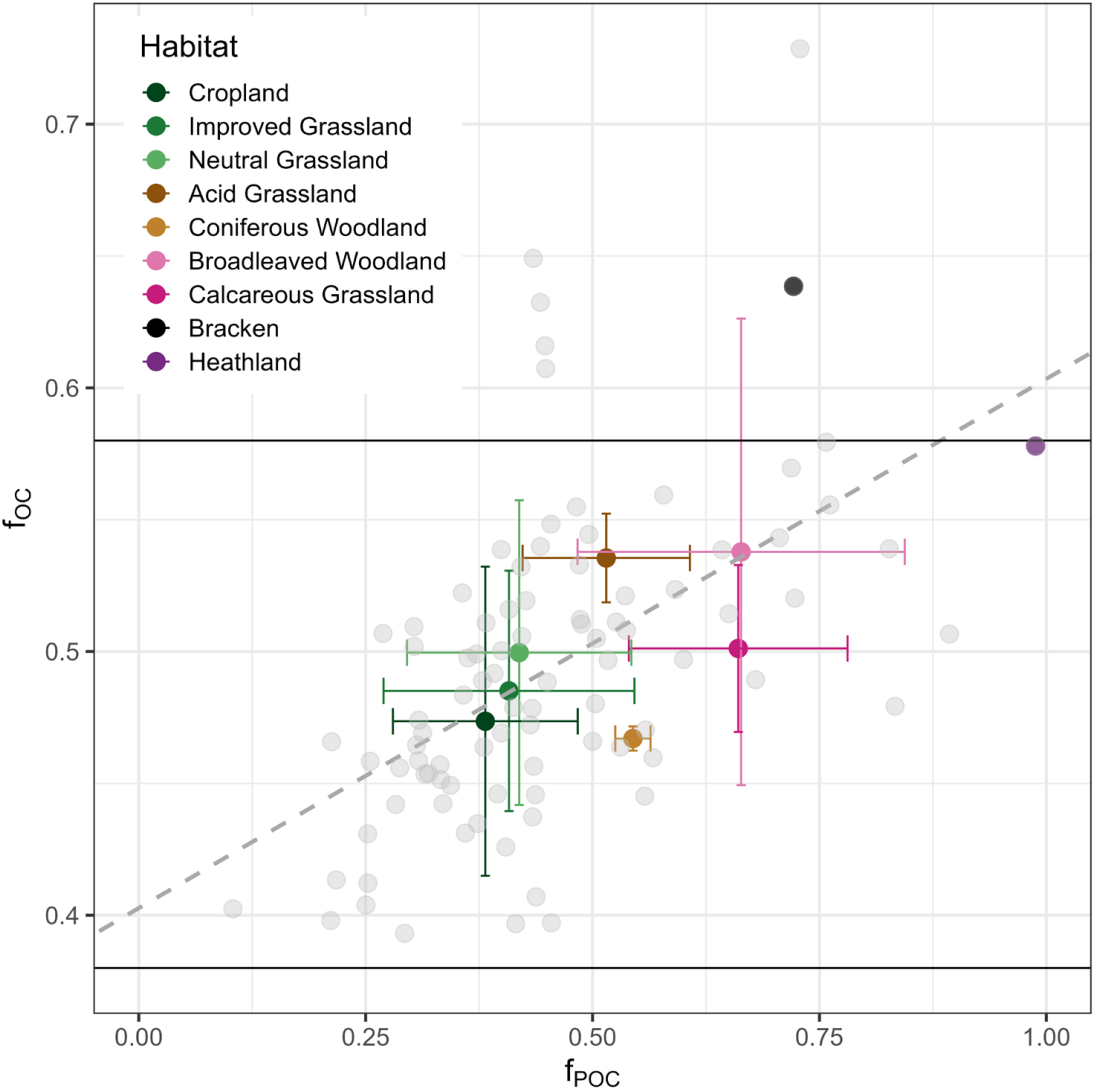
The fraction of organic carbon in soil organic matter, $f_{\mathrm{OC}}$, correlated with the fraction of particulate organic matter carbon in SOC [*r*(90) = 0.54, *p* < 0.001], $f_{\mathrm{POC}}$, across various habitats. Horizontal lines indicate $f_{\mathrm{OC}}$ values of 0.38 and 0.58, which correspond to the average carbon content of crop roots (38%) and humic acid (58%), respectively. The dashed grey line illustrates the linear regression applied to the raw data points shown in grey. The dataset is biased in numbers, with highest sample numbers for cropland (*n* = 33), improved and neutral grasslands, and lowest numbers for coniferous woodland (*n* = 2), bracken (*n* = 1) and heathland (*n* = 1) (Table S3; Figure S4).

In accordance with the ‘consolidated view’ presented by Lehmann and Kleber (2015), it is expected that POC, primarily unprocessed SOM, and MAOC would establish a continuum in relation to $f_{\mathrm{OC}}$ ranging from approximately 0.38 to 0.58 for mineral soils. Using a subset of the UKCEH Countryside Survey data (Lebron et al. 2025) across various habitats, an analysis was conducted on POM and MAOM to determine their carbon contents alongside SOC and SOM. As illustrated in Figure 6, higher $f_{\mathrm{OC}}$ values are associated with the POC fraction of SOC $\left(f_{\mathrm{POC}}\right)$, thereby supporting the concept of a thermodynamic gradient. In croplands, neutral and improved grasslands, less than 50% SOC is linked to POM (Figure 6). Approximately 50% of the SOC in coniferous woodland and acid grassland is associated with POM and MAOM, respectively, with coniferous woodlands storing considerably less SOC in POM compared to acid grasslands. In broadleaved woodland and calcareous grasslands, over 50% of SOC is retained as POC, while the majority of SOC in heathland soil is associated with POM (Figure 6). The data is summarised in Table S3.

## 4 Discussion

We describe $f_{\mathrm{OC}}$ as the amount of SOC per unit SOM. The data points fall roughly between slopes of 0.38 and 0.58, with an overall average slope of 0.54, which is more aligned with carbon in processed organic matter than in ‘unprocessed’ organic matter, as illustrated in Figure 1. The data support the ‘consolidated view’ presented in the soil continuum model by Lehmann and Kleber (2015) (Basile‐Doelsch et al. 2020; Lehmann and Kleber 2015), indicating a gradual decomposition of SOM resulting from vegetation inputs, with the levels of $f_{\mathrm{OC}}$ determined by the physical properties of the soil (Figure 4).

The categorisation of soil, especially regarding the presence of habitats on either mineral or organic soil, plays a crucial role in determining the baseline of $f_{\mathrm{OC}}$. The greater accumulation of SOC relative to SOM within organic soils is likely due to the long‐term decomposition of SOM, leading to a reduction in structural complexity and an increase in carbon concentration. This is illustrated in Figure S3 (Van Krevelen 1950), which demonstrates how dehydration and decarboxylation processes remove bound water (H and O) and hydroxyl (OH) groups, thereby making nitrogen and carbon accessible to the soil environment. Unlike organic soils, carbon in mineral soil may primarily occur in dissolved form and is less processed due to its protection from decomposition on mineral surfaces. The two sources of MAOC, combined with the protection against degradation (Rocci et al. 2024; Six et al. 2002) and a potential limitation of exchangeable clay mineral sites (Georgiou et al. 2025), contribute to a generally lower $f_{\mathrm{OC}}$ in mineral soils when compared to organic soils.

Furthermore, the data from the UKCEH Countryside Survey national dataset indicate a potential correlation between POC and $f_{\mathrm{OC}}$ that warrants further investigation at broader scales, particularly regarding their reliance on soil physical properties. Figure 6 reinforces the idea that a linear relationship exists between the $f_{\mathrm{OC}}$ and POC and MAOC fractions of SOM, as one might expect from the soil continuum model. POC is linked to predominantly unprocessed inputs and large biopolymers, while MAOC is associated with processed material and smaller biopolymers or monomers that are adsorbed onto mineral surfaces. The linear relationship shown between 0.38 and 0.58 in Figure 6 is illustrative and will not be universal; variations in foc are likely as the carbon content of organic matter inputs are influenced by plant inputs (Figure S2), habitat composition (Ma et al. 2018) and soil properties (Figure 4). The correlation between $f_{\mathrm{POC}}$ and $f_{\text{MAOC}}$ with $f_{\mathrm{OC}}$ suggests that it may be possible to extrapolate from a limited number of POC and MAOC measurements by using the more commonly available SOC and SOM metrics. This supports the argument for the inclusion of both SOC and SOM in regular monitoring programs, as it could provide an initial estimate of POC and MAOC based on $f_{\mathrm{OC}}$.

### 4.1 Variability within $f_{\mathrm{OC}}$

We show that over 50% of the variability in $f_{\mathrm{OC}}$ can be attributed to habitat and soil category (along with its association with SOC) alone. The explanatory power increases to 74% when soil profile identity is incorporated into the statistical model. There is a considerable scatter of $f_{\mathrm{OC}}$ within habitat, primarily explained by soil category, where soil properties determine the attainable level of $f_{\mathrm{OC}}$ (Figure 4), with habitat influencing the thresholds established by each soil category. The variations in $f_{\mathrm{OC}}$ are likely due to multiple processes operating across different scales (Wiesmeier et al. 2019). These processes may be linked to species‐specific carbon contents in both aboveground and belowground plant organic matter (Cools et al. 2014; Ma et al. 2018), in conjunction with environmental constraints, habitat responses to climate change, land management or land‐use change, and changes resulting from policy interventions aimed at enhancing SOC stocks (particularly in cropland soils) (De Rosa et al. 2024; Emmett et al. 2023).

If the degree of organic matter processing significantly affects $f_{\mathrm{OC}}$, it is expected that a latitudinal gradient will exists. This relationship may indicate a slower degradation of SOM in the cooler Northern latitudes, along with a prevalence of relatively unprocessed POM in topsoils, until SOM undergoes biogeochemical alteration (Cotrufo et al. 2019; Schuur et al. 2022) (Figure S8). While distinct datasets show variations in $f_{\mathrm{OC}}$ that could also be linked to latitude and temperature differences, the impact of habitat on $f_{\mathrm{OC}}$ is clear (Figure 4; Figures S8 and S9) (Heikkinen et al. 2022; Plaza et al. 2019; Taghizadeh‐Toosi et al. 2014; van Huissteden and Dolman 2012). Considering the significant SOC losses anticipated from the thawing of permafrost and the drying of peatlands (Schuur et al. 2022; Swindles et al. 2019), the overall global changes in SOC resulting from rising temperatures, changed precipitation patterns, and increased atmospheric CO_2_ concentrations are comparably small when compared to the impacts of land‐use changes and land management practices that actively manipulate SOM contents (Guo and Gifford 2002; Poeplau et al. 2015). Notable exceptions occur in scenarios where warming leads to melting of ice or, exposing soils (Plaza et al. 2019; van Huissteden and Dolman 2012), or when peatlands dry out (Swindles et al. 2019).

There are several examples where policy interventions have directly impacted SOC stocks and $f_{\mathrm{OC}}$. Poeplau et al. (2015) found, due to social‐economic incentives, leys were cultivated on agricultural land in Sweden, leading to an annual increase in topsoil SOC content of 0.38%. Such incentives have the potential to increase $f_{\mathrm{OC}}$ across the habitat gradient illustrated in Figures 3 and 4. For example, by incorporating SOM into cropland soils, new SOC will be introduced into the soil system, thereby increasing $f_{\mathrm{OC}}$, particularly when organic matter rich in organic carbon is added. A different study concerning Finnish soils reported a reduction in SOC content within the top 0–10 cm of boreal topsoil from 2009 to 2018, during which no policy incentives were in effect (Heikkinen et al. 2022). However, SOC depletion was mitigated in Finnish cultivated soils due to land management practices that counteracted the adverse climate effects. These examples demonstrate that through meticulous management of SOM, landowners can enhance $f_{\mathrm{OC}}$, leading to beneficial outcomes for soil health. Conversely, in the EU and UK, an analysis of approximately 5700 repeated points of the LUCAS surveys in 2009 and 2018 across arable lands, De Rosa et al. (2024) showed a 0.75% decline in SOC stock over a 9‐year time span, suggesting that policy incentives for SOC management have yet to be effectively implemented at the European level.

### 4.2 $f_{\mathrm{OC}}$ by Habitat

The impact of habitat on $f_{\mathrm{OC}}$, once the soil type (or category) determines the level of soil carbon storage capacity (Figure 4), enables us to understand the potential of land use and management to enhance SOC storage. Considering the positive relationship between carbon and lignin content (but not with cellulose content) (Ma et al. 2018), we expect variations in $f_{\mathrm{OC}}$, particularly between herbaceous and woody plants. This is consistent with the life strategies of woody species, which allocate more resources to structural compounds such as lignin to enhance their longevity and resistance to decomposition, while herbaceous plants, which generally have shorter life spans, prioritise faster growth with lower lignin content. Furthermore, the investment of carbon in plant structural components is reduced in ocean vegetation, as the water column aids in maintaining their upright position.

Habitats characterised by herbaceous vegetation (not limited by pH) show lower predicted $f_{\mathrm{OC}}$ values in comparison to woody vegetation (Figure 4), even when considering soil category. Croplands, neutral and improved grasslands display equivalent levels of $f_{\mathrm{OC}}$, likely due to the generally lower leaf and root carbon contents compared to woody vegetation (Ma et al. 2018) (Figure S2). In cropland systems, it is anticipated that biomass turnover, productivity, and potential disturbance from land management practices will be high. The combination of low SOM contents and soil management in croplands, along with herbaceous litter input, leads to the depletion of the occluded POC fraction of SOM (Willard et al. 2024). In neutral and improved grasslands, SOC and SOM contents are approximately twice that of croplands (Table S2). Given that the conversion between (improved) grasslands and croplands is common practice, it is not unexpected that land‐use change results in the most significant loss of SOC globally (Beillouin et al. 2023). In a similar study, De Rosa et al. (2024) demonstrated that the primary cause of carbon loss in the EU and UK from 2009 to 2018 was the conversion of grassland into cropland. Interestingly, land management practices frequently exhibited a beneficial impact on SOC stocks (Beillouin et al. 2023), highlighting the potential of targeted land (and soil) management. Nevertheless, these findings need further exploration concerning $f_{\mathrm{OC}}$, as the effects of grazing or grassland management are studied rather than considering SOC and SOM collectively over time. Based on our findings, future research could focus on the variations in $f_{\mathrm{OC}}$ associated with land‐use change (as opposed to habitat) to uncover the extent to which the variability observed within each habitat is linked to changes in land use, and whether this is influenced by an accumulation of SOM or a reduction in SOC.

Data on SOC in (semi‐) natural habitats such as heathland, bracken, neutral and acid grasslands is less comprehensive compared to the primary habitats found throughout Europe, including cropland, grasslands and woodlands (Tóth et al. 2013). Our $f_{\mathrm{OC}}$ indicator for natural systems indicates that the absence of soil disturbance, the presence of natural plant communities, and effective grazing management contribute to higher $f_{\mathrm{OC}}$ values (Figures 3 and 4). It is likely that moderate disturbance from grazing, along with continuous input of aboveground and the turnover of the rooting system, help sustain high SOM levels for the soil microbial community to use. Considering the significance of soil microorganisms in the formation of MAOC (Lavallee et al. 2020), the carbon storage in MAOM within natural systems on mineral and organo‐mineral soils may be improved when transitioning from intensively to less managed habitats. Taking into account microbial stoichiometry, it is probable that natural habitats will display $f_{\mathrm{OC}}$ values more aligned with those of the microbial community, as SOM is processed to decrease stoichiometric imbalances (Mooshammer et al. 2014). At this point, this remains a theoretical consideration that is based on the carbon contents of *E. coli* and *M. bicolor* (refer to Figure 1) as well as the $f_{\mathrm{OC}}$ values observed for (semi‐) natural systems, which are approximately 0.5 or higher (see Figure 3, orange and brown). Furthermore, heathland systems are dominated by ericoid mycorrhiza associations, which affects the cycling of carbon and nutrients in both soil and microbial communities. Similar to coniferous woodlands, the presence of ericoid mycorrhiza leads to higher SOC contents per unit of N, thereby likely increasing $f_{\mathrm{OC}}$ (Cotrufo et al. 2019).

Interestingly, Peatlands exhibit predicted $f_{\mathrm{OC}}$ values that are comparable to these of herbaceous vegetation. However, it is reasonable to conclude that distinct processes lead to these similar $f_{\mathrm{OC}}$ values: as indicated in Figure 5, the decomposition of SOM in peatlands is not the sole factor affecting the equilibrium of $f_{\mathrm{OC}}$, which is also influenced by land use and/or drainage practices applied to peat. The long‐term stabilisation of SOM may play a pivotal role in influencing Peatland SOM when soils are oxygenated and SOC is lost due to increased microbial activity, leading to alterations in SOM stoichiometry (Leifeld et al. 2020) and thus densities (Figure S3).

### 4.3 $f_{\mathrm{OC}}$ in Woodlands

For different types of woodlands, $f_{\mathrm{OC}}$ indicates the presence of distinct soil processes (Figure 3). The variation in $f_{\mathrm{OC}}$ between broadleaved and coniferous woodlands appears to be related to the differing SOM contents (Figure 4) and the types of soil on which these forests are primarily planted. Broadleaved woodlands are typically planted on heavier and richer soils, whereas coniferous woodlands are more commonly planted on organic soils which influences the fraction of SOM available for SOC storage. Coniferous woodlands have an average SOM content of 3.7 g kg^−1^ soil, approximately three times greater than the average SOM content for broadleaved woodlands (1.3 g kg^−1^). This increased SOM content in coniferous woodlands correlates with higher SOC contents (2.0 g kg^−1^), leading to overall higher $f_{\mathrm{OC}}$ values (0.53, Table S2). Additionally, the soil category and nutrients, along with SOM contents, are complemented by the composition of the soil fungal community (Awad et al. 2019), which affects SOC stocks in the woodland organic horizon (Anthony et al. 2024). This indicates that the fungal community exerts a greater impact on $f_{\mathrm{OC}}$ if planted on organic soils.

Saprophytic fungi favour lower soil pH levels and degrade organic matter to obtain carbon, while ectomycorrhizal fungi engage in nutrient exchange for carbon derived from plants. Temperate coniferous woodlands have up to 54% higher fungal biomass in comparison to pure broadleaved woodlands. (Awad et al. 2019). Furthermore, it was found that saprophytic fungi in pure temperate coniferous woodlands were twice as abundant as those in pure broadleaved woodlands. Interestingly, this advantageous relationship in coniferous woodlands was not seen for ectomycorrhizal fungi. With a more active saprotrophic fungal community, coniferous woodland releases more SOC through direct respiration than broadleaved woodland, as noted in Moyano et al. (2008). However, despite the higher respiration rates, the higher SOM content in coniferous woodland prevents a decrease in $f_{\mathrm{OC}}$.

The biomass of saprotrophic and ectomycorrhizal fungi is influenced by the composition of forest tree species (Awad et al. 2019), suggesting that fungi may significantly contribute to the variations in $f_{\mathrm{OC}}$ in woodlands of this study. Additionally, there are other distinctions between coniferous and broadleaved woodlands, such as rates of phenol leaching (Kuiters 1990; Kuiters and Sarink 1986) and variations in root and fine root properties and turnover times (Finér et al. 2007; Förster et al. 2021) which also impact the persistent difference in $f_{\mathrm{OC}}$ across woodland types.

### 4.4 $f_{\mathrm{OC}}$ in Sediments and Permafrost Regions

Yedoma and Seagrass sediments exhibit the lowest predicted $f_{\mathrm{OC}}$ values (Figure 4), likely due to the comparatively low input of organic matter in permafrost sediments and the water column, respectively. Seagrass sediments have demonstrated a significant, potentially unlimited capacity for carbon sequestration (European Environment Agency 2022). In contrast, in Seagrass sediments [and saltmarshes (Maxwell et al. 2023)], there is a substantial influx of allochtonous organic matter (Temmink et al. 2022), and the rate of decomposition is reduced with increasing salinity in porewater (Ouyang et al. 2017). In these environments, the content of SOM is high (1.5 g kg^−1^), yet the $f_{\mathrm{OC}}$ values are relatively low, possibly due to high concentrations of inorganic carbon and low carbon in incoming organic matter (Figure S2).

The $f_{\mathrm{OC}}$ of Yedoma sediments represents the lower range of values found in Permafrost habitats, likely associated with the soil properties of these Pleistocene sediments, which are distinctly different from Permafrost soils (Shur et al. 2022). It has been proposed that Yedoma soils contain approximately 2.6% SOC or less (Strauss et al. 2017), which is close to the median SOC value of 1.9% observed in this study (Table S2). In Permafrost regions, the production of organic matter, along with the formation and decomposition of SOM, is constrained by temperature and ground ice (Schuur et al. 2022), as well as the absence of major macrofauna species, such as earthworms, in Arctic environments (Makarova and Kolesnikova 2019; Wackett et al. 2018). The low $f_{\mathrm{OC}}$ values result from relatively low SOC values (median 0.2 g kg^−1^) in comparison to SOM (median 0.6 g kg^−1^), which can be explained by seasonal SOC loss as dissolved organic carbon during permafrost thawing (Liu et al. 2022). With the intensification of global warming, it is anticipated that carbon emission from Permafrost soils will reach 55 Pg C‐CO_2_‐e to 232 Pg C‐CO_2_‐e by the year 2100 (Schuur et al. 2022), resulting in a reduction of Permafrost SOC concentration and stock. Nevertheless, as temperatures in Arctic systems increase, vegetation simultaneously adopts, a potentially mitigating SOC loss by increasing organic matter (Natali et al. 2012) and leading to unpredictable effects on $f_{\mathrm{OC}}$. Monitoring $f_{\mathrm{OC}}$ across Permafrost systems may identify areas of overall ecosystem stability (indicated by stable $f_{\mathrm{OC}}$ values) or degradation (characterised by decreasing $f_{\mathrm{OC}}$ values), thus identifying areas in need of land management interventions.

### 4.5 Carbon Storage in SOM Fractions

Considering the good relationship between $f_{\mathrm{OC}}$, habitats and soil categories, we explored whether our indicator could serve as a predictor for carbon stored in POM and MAOM fractions. If this is the case, the conventional metrics of SOC and SOM may offer a cost‐effective and spatially available alternative for estimating POC and MAOC on national and continental scales. Such estimates could play a crucial role in guiding essential policy decision aimed at enhancing soil management strategies.

Previous research has demonstrated consistent trends of $f_{\mathrm{OC}}$ in SOM fractions. Pulido‐Moncada et al. (2018) compared the relationship between SOC and SOM fractions (along other soil physical characteristics) in Belgian and Venezuelian soils subjected to different land uses. The soils from these two countries had very different SOC contents yet indicated that SOC and SOM were affected by land use. Furthermore, land use was considered crucial for understanding SOM fractions in studies conducted by Cotrufo et al. (2019) and Lugato et al. (2021), who analysed the European LUCAS dataset concerning SOM fractions across different land uses. Additionally, Willard et al. (2024) observed that land uses account for the distribution of carbon within SOM fractions.

In accordance with the understanding that land use, and consequently habitat, influences SOC in SOM fractions, we used a subset of the Countryside Survey topsoil data (0–15 cm) that includes $f_{\mathrm{OC}}$, MAOC and POC (Lebron et al. 2025). This analysis allowed us to explore the relationship between $f_{\mathrm{OC}}$ and SOC stored in the two SOM fractions. As reported by Wiesmeier et al. (2019), the storage of SOC at larger scales is influence by factors such as climate, vegetation or land use, and the soil parent material. It is reasonable to expect that the storage of SOC in SOM fractions at regional or continental levels would be similarly affected by the same drivers (Lavallee et al. 2020; Zhou et al. 2024). The variations in POC accumulation at the continental level is primarily driven by climate and its effects on plants, while the accumulation of MAOC is frequently referred to soil properties (such as clays, silt, cation‐exchange capacity) and the activity of soil microorganisms (Cotrufo and Lavallee 2022; Lavallee et al. 2020).

Cropland soils, with the exception of crop production on peat, along with grasslands, retain between 50% and 70% of SOC in the form of MAOC (Bai and Cotrufo 2022). Consequently, for these habitats, the SOC stored as POC will consistently be at the lower range. Referring to Figure 6 for cropland, improved, and neutral grasslands, the average $f_{\mathrm{POC}}$ is approximately 40%, which corresponding $f_{\mathrm{OC}}$ values reaching up to 0.5. These habitats represent the lower end of a promising $f_{\mathrm{POC}}$ gradient established from $f_{\mathrm{OC}}$. The majority of our data points concerning SOM fractions fall into these habitats (Table S3; Figure S4).

The following two habitats along the $f_{\mathrm{POC}}$ gradient are acid grassland and coniferous woodland, both of which are characterised by low soil pH levels. When compared to the entire dataset, coniferous woodland shows a distinctly lower $f_{\mathrm{OC}}$, which may be coincidental as it falls at the lower end of magnitude of $f_{\mathrm{OC}}$ for that particular habitat (refer to Table S2; Figure S4). Conversely, the $f_{\mathrm{OC}}$ value of acid grassland soil reflects the characteristics of the full dataset; its POC loss is likely primarily influenced by microbial limitations due to the low soil pH, resulting in less processed SOM that remains in the soil as POC (Malik et al. 2018). This is because POM must undergo processing before POM‐derived carbon can bind with soil minerals (Cotrufo and Lavallee 2022).

Following along the $f_{\mathrm{POC}}$ gradient, broadleaved woodland and calcareous grassland soils are next. In broadleaved woodlands, pH is not likely to be the limiting factor for the microbial decomposition of SOM; however the structure of roots and microbial associations influence the degradation of POM, resulting in the accumulation of POC. Furthermore, the quantity of plant biomass, along with faunal and microbial necromass entering the soil system, is significantly higher than that found in grasslands (Kögel‐Knabner 2002), which likely delays the degradation of POM. In the case of calcareous grasslands, soil pH may again play a crucial role in SOC storage, with microbial limitations arising from high soil pH (Bolan et al. 2023; Yan et al. 2022) and the storage of inorganic carbon on minerals.

Finally, the upper range of the $f_{\mathrm{POC}}$ gradient is defined by semi‐natural systems (bracken and heathland) featuring slow‐growing plants, primarily constrained by low temperatures and high soil moisture levels. Temperature and the chemical composition of the vegetation that enters the soil can influence the degradability of organic matter (Crowther et al. 2016; Kögel‐Knabner 2002; Lehmann and Kleber 2015). In upland systems, the decomposition of SOM is limited by high water tables, resulting in anoxic conditions, which defines the upper range of the $f_{\mathrm{POC}}$ gradient.

## 5 Conclusions

The fraction of organic carbon in SOM, denoted as $f_{\mathrm{OC}}$, offers valuable insight into the dynamics of soil processes that extend beyond the evaluation of each individual metrics. The fraction is influenced by plant inputs and consequently varies with habitat, being further regulated by microorganisms and macrofauna prevalent across major soil categories. We propose that $f_{\mathrm{OC}}$ serves as a reliable indicator of long‐term changes (e.g., over 5–10 years) that are relevant to policy‐making by mitigating the effects of land use and environmental changes (such as temperature, moisture, pH, etc.) (FAO 2020). The implementation of national or continental monitoring of $f_{\mathrm{OC}}$ across various habitats and soil types as a rolling survey, which involves sampling a representative subsample over multiple years rather than a single year, will also help to minimise the bias introduced by annual variability (e.g., extreme heat) on ecosystem processes (Robinson et al. 2024). On a continental scale, the levels of $f_{\mathrm{OC}}$ are expected to be affected by temperature and rainfall (e.g., Figure S8); however, the direct impacts of these factors cannot be isolated from our dataset due to the unavailability of geo‐referenced sampling information. By integrating $f_{\mathrm{OC}}$ into soil monitoring initiatives, policymakers would be able to more precisely identify areas with high SOC storage potential and develop more focused carbon sequestration strategies. This may include improving agricultural soil management, refining land‐use planning (e.g., woodlands), and tracking climate‐related SOC targets.

In addition, a novel analysis of a subset of UK data shows a remarkably close relationship between $f_{\mathrm{OC}}$, habitats and the SOM fractions POC and MAOC (Figure 6). This relationship offers the potential to improve predictions of SOM fractions in a spatial context, as demonstrated by Zhou et al. (2024). Additionally, the more readily accessible SOC and SOM measurements can be used to derive our $f_{\mathrm{OC}}$ metrics, allowing us to explore it as a covariate for the prediction of SOM fractions. Exploitation of this relationship may facilitate a more refined spatial understanding of POC and MAOC, respectively. Another possible application of $f_{\mathrm{OC}}$ and the relationship between carbon fractions and habitats is found in processed‐based modelling. According to Sulman et al. (2018), processed‐based models, even when provided with the same data, generate significantly different predictions regarding SOC dynamics. Our indictor, if used to differentiate SOC into POC and MAOC across various habitats and soil types, could contribute to a harmonisation of process‐based modelling methodologies by consistently deriving stable (MAOC) and labile (POC) components in a consistent manner across models. By implementing the theoretical constraints of $f_{\mathrm{OC}}$ as illustrated in Figure 1, SOC saturation is fundamentally governed by the data, which would mitigate the uncertainty in SOC process modelling, as highlighted by Georgiou et al. (2025) as an area for future investigation.

Major questions remain concerning the variations in $f_{\mathrm{OC}}$ attributed to land‐use changes or latitude, as well as the changes resulting from differences in mean annual precipitation and temperature. When we categorise our data broadly into Northern and Southern countries, there appears to be a general trend of lower $f_{\mathrm{OC}}$ values at higher latitude, as evidenced through POC and MAOC (Zhou et al. 2024). However, a more detailed examination based on locations is necessary, which was not feasible in this study (Figure S8). This analysis will not only enhance our understanding of SOC and SOM dynamics but may also uncover innovative strategies to address the significant environmental challenges society faces.

In the future, when geo‐referenced and methodologically consistent data (such as LUCAS) become available, we will be able to investigate the relationships of $f_{\mathrm{OC}}$ in relation to temperature and precipitation gradients, along with other environmental co‐variates such as soil texture. While national soil monitoring programmes may not be directly associated with land management and policy incentives, the measurement of SOC and SOM can, in the future, serve as a benchmark for evaluating current and potential soil management practices across various habitats (Feeney et al. 2023, 2025).

## Author Contributions

**Sabine Reinsch:** conceptualization, data curation, formal analysis, methodology, writing – original draft, writing – review and editing. **Inma Lebron:** conceptualization, methodology, writing – original draft, writing – review and editing. **Lis Wollesen de Jonge:** funding acquisition, writing – original draft. **Peter L. Weber:** formal analysis, methodology, writing – original draft. **Trine Norgaard:** methodology, writing – original draft. **Emmanuel Arthur:** methodology, writing – original draft. **Lucas Gomes:** methodology, writing – original draft. **Charles Pesch:** methodology, writing – original draft. **Karyotis Konstantinos:** data curation, methodology, writing – original draft. **George Zalidis:** methodology, writing – original draft. **Lur Epelde:** data curation, funding acquisition, methodology, writing – original draft, writing – review and editing. **Marija Romic:** data curation, methodology, writing – original draft. **Davor Romic:** methodology, writing – original draft. **Monika Zovko:** methodology, writing – original draft. **Marko Reljic:** methodology, writing – review and editing. **Jaakko Heikkinen:** formal analysis, methodology, writing – original draft. **Peter Levy:** conceptualization, formal analysis, methodology, validation, writing – original draft. **Elena Vanguelova:** data curation, methodology, writing – original draft, writing – review and editing. **Panos Panagos:** methodology, writing – original draft. **Florian Schneider:** investigation, writing – original draft. **Bernhard Ahrens:** writing – original draft. **Jens Leifeld:** writing – original draft. **Gustaf Hugelius:** methodology, writing – original draft. **Christopher Feeney:** formal analysis, writing – original draft. **Laura Bentley:** funding acquisition, writing – original draft. **Bridget A. Emmett:** funding acquisition, writing – original draft. **Bernhard J. Cosby:** funding acquisition, writing – original draft. **Michele Brentegani:** methodology, writing – original draft. **Susan Tandy:** methodology, writing – original draft. **Amy Thomas:** writing – original draft. **Maud A. J. van Soest:** writing – original draft. **David A. Robinson:** conceptualization, formal analysis, funding acquisition, methodology, validation, visualization, writing – original draft, writing – review and editing.

## Disclosure

Work funded by the European Union. Views and opinions expressed are, however, those of the authors only and do not necessarily reflect those of the European Union or the Research Executive Agency. Neither the European Union nor the granting authority can be held responsible for them.

## Conflicts of Interest

The authors declare no conflicts of interest.

## Supporting information


**Data S1:** gcb70572‐sup‐0001‐DataS1.xlsx.

**Data S2:** gcb70572‐sup‐0002‐DataS2.docx.

## Data Availability

The data that support the findings of this study are openly available in Zenodo at https://doi.org/10.5281/zenodo.17305831. Soil organic matter fractionation data are freely available on the NERC EDS Environmental Information Data Centre at https://doi.org/10.5285/29cd5386‐bc2e‐4d70‐a6a5‐0d7dc7513dc6.
