# Supplementary material for: The Fraction of Carbon in Soil Organic Matter as a National‐Scale Soil Process Indicator
Source: Glob Chang Biol. 2025 Oct 30;31(11):e70572. doi: 10.1111/gcb.70572 (PMC12573071; doi:10.1111/gcb.70572)
Supplement: Supplementary file 2 — Data S2: gcb70572‐sup‐0002‐DataS2.docx. [file GCB-31-e70572-s001.docx]

**The fraction of carbon in soil organic matter as a national-scale soil process indicator**

**Running Title:** fraction of carbon in soil organic matter

**List of Authors**: Sabine Reinsch^1*^, Inma Lebron^1^, Lis Wollesen de Jonge^2^, Peter L. Weber^2^, Trine Norgaard^2^, Emmanuel Arthur^2^, Lucas Gomez^2^, Charles Pesch^2^, Karyotis Konstantinos^3^, George Zalidis^3^, Lur Epelde^4^, Marija Romic^5^, Davor Romic^5^, Monika Zovko^5^, Marko Reljic^5^, Jaakko Heikkinen^6^, Christopher Feeney^1^, Laura Bentley^1^, Peter Levy^7^, Elena Vanguelova^8^, Panos Panagos^9^, Florian Schneider^10^, Bernhard Ahrens^11^, Jens Leifeld^12^, Gustaf Hugelius^13^, Bridget A. Emmett^1^, Bernhard J. Cosby^1^, Michele Brentegani^1^, Susan Tandy^1^, Amy Thomas^1^, Maud A.J. van Soest^1^, David A. Robinson^1^

**Institutional affiliations**

^1^ UK Centre for Ecology & Hydrology, Deiniol Road, Bangor, United Kingdom

^2^ Aarhus University, Department of Agroecology, Tjele, Denmark

^3^ Laboratory of Remote Sensing, Spectroscopy, and GIS, Department of Agriculture, Aristotle University of Thessaloniki, Thessaloniki, Greece

^4^ NEIKER-Basque Institute for Agricultural Research and Development, Derio, Spain

^5^ University of Zagreb Faculty of Agriculture, Zagreb, Croatia

^6^ Natural Resources Institute, Jokioinen, Finland

^7^ UK Centre for Ecology & Hydrology, Bush Estate, Penicuik, United Kingdom

^8^ Forest Research, Farnham, United Kingdom

^9^ European Commission, Joint Research Centre (JRC), Ispra (VA), Italy

^10^ Thünen Institute of Climate-Smart Agriculture, Braunschweig, Germany

^11^ Max Planck Institute for Biogeochemistry, Jena, Germany

^12^ Agroscope, Climate and Agriculture Group, Zurich, Switzerland

^13^ Department of Physical Geography and Bolin Centre for Climate Research, Stockholm Sweden

Supplementary information to “***The fraction of carbon in soil organic matter as a national-scale soil process indicator***” by Reinsch et al.

**Dataset habitats and distribution**

Given the potential relevance of the relationship between soil organic carbon (SOC) and soil organic matter (SOM) in the EU’s mission for measurable soil health indicators (Panagos et al., 2022), we searched the literature and contacted partners within the European AI4SoilHealth project from areas with highly contrasting environmental characteristics (including climate and land management intensity) to robustly test for a relationship between SOC and SOM. These included data for Cropland soils (Finland, UK), Woodlands (Spain, Permafrost regions, UK), Grasslands (Permafrost regions, Spain, UK), Barren, Tundra and Yedoma (Permafrost regions), Semi-natural habitats such as Bracken and Heathland (Permafrost regions, UK), Peatlands (Switzerland, UK), and Seagrass (global) (Supplementary Fig. 1). An overview of datasets and methods is provided in the Supplementary Table 1 below. The dataset is available from Reinsch, Weber, et al. (2025).


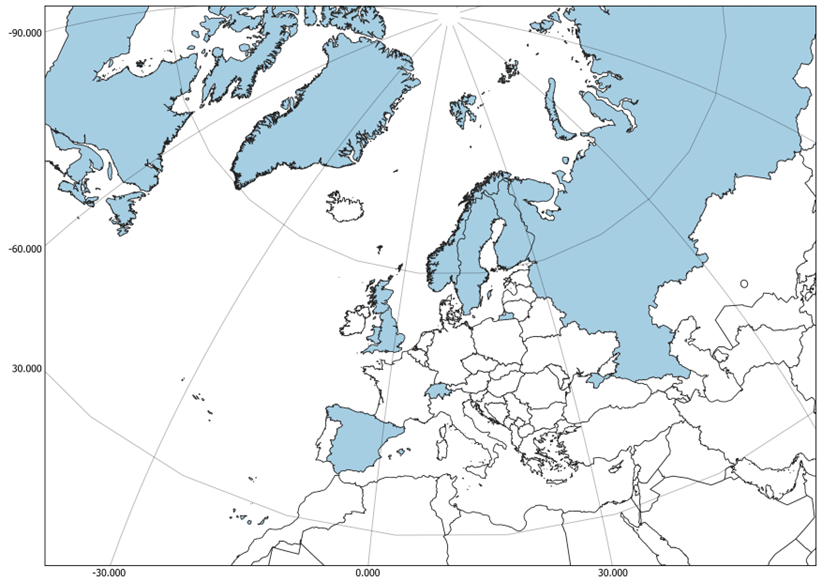


**Supplementary Fig. 1** Overview of countries covered in this data analysis including data from Temperate and Permafrost regions. The distribution of global Seagrass data points is shown in Fourqurean et al. (2012).


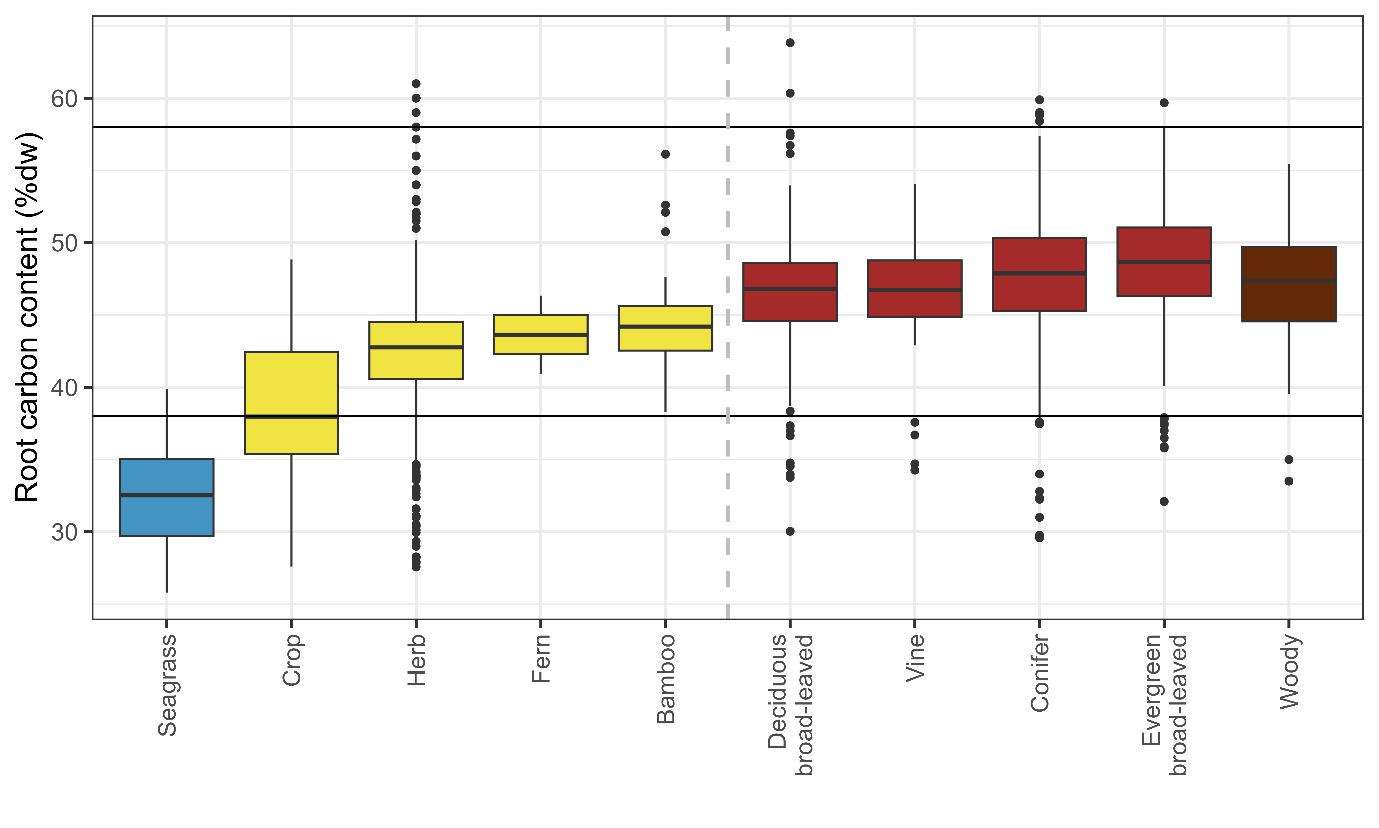


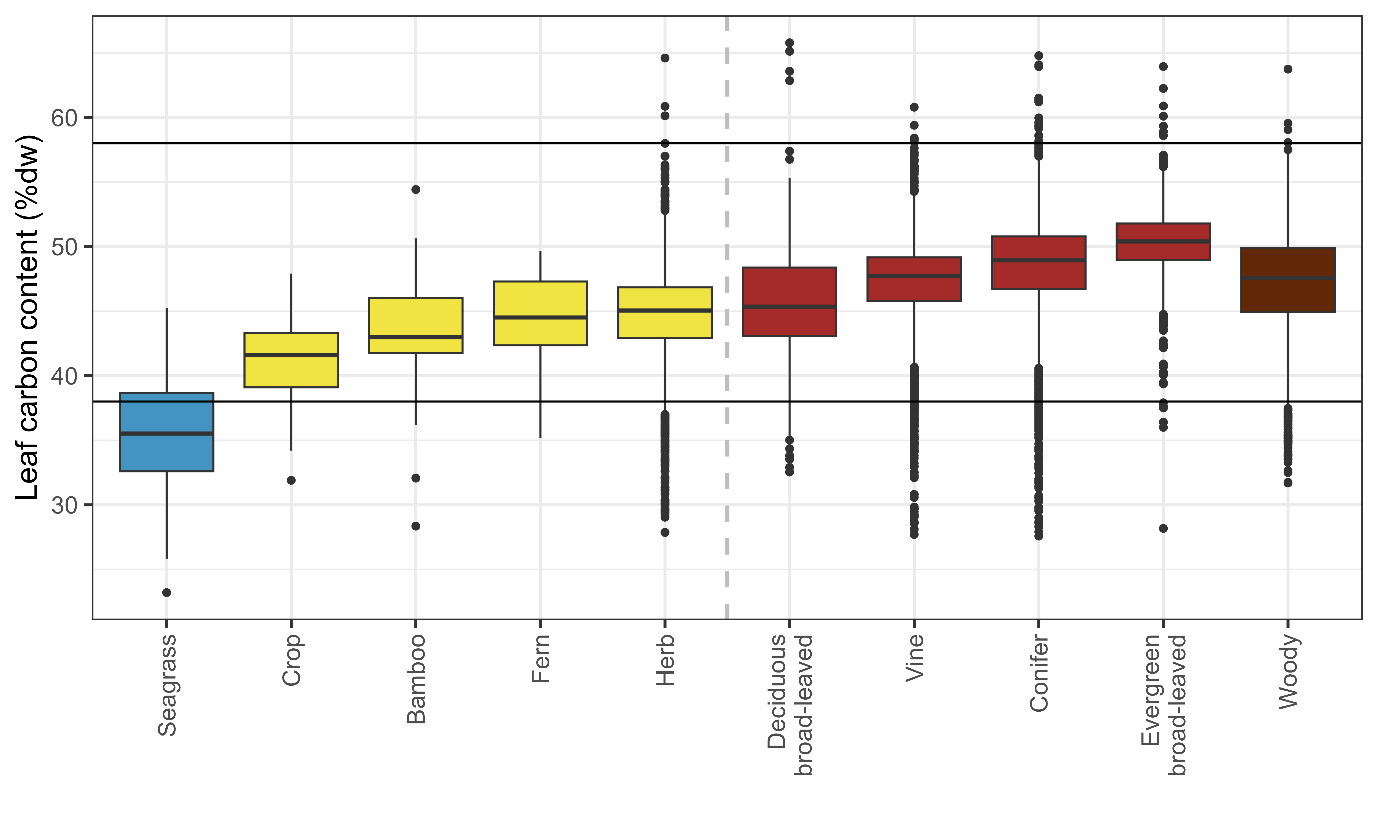


**Supplementary Fig. 2** Root (top) and leaf (bottom) carbon contents (% carbon in biomass) calculated from data by (Ma et al., 2018); Organic carbon of Seagrass roots and rhizomes were added from the literature (Holmer et al., 2004; Jiang et al., 2019; Luo et al., 2024); Blue: Seagrass, Yellow: herbaceous plants or habitat, brown: woody plants or woodlands, dark brown: general category of woody plants where the life form was not specified. The two black lines correspond to the average crop root carbon content (38%) and carbon content in humic acid (58%) in line with Fig. 1.

**Van Krevelen diagram**

The habitat effect on $f_{OC}$ manifests through habitat-specific plant inputs to soils with an impact on the initial chemical composition of SOM which affects its decomposition rate. Biomass ‘quality’ can be assessed using information on oxygen-to-carbon (O:C) and hydrogen-to-carbon (H:C) measured on SOM to determine the heating value of biomass (Van Krevelen, 1950). By synthesising data from plant roots, through peats to coals, a Van Krevelen diagram can be produced providing insight into organic matter ‘quality’ for organic soils. We produced a Van Krevelen diagram (Supplementary Fig. 3) based on Peatland data from Leifeld et al. (2020) and fuel data from Ahmad & Subawi (2013).

O:C ratios vary from as low as 0.02 to as high as 1, with the mean crowd of data ranging between 0.3 and 0.8. The very low and very high O:C values were for crops and grass (growing on peat) only. However, there was no separation of land uses on natural peat in O:C values. H:C ratios were used to determine the ‘maturity’ of the organic matter with hydrocarbons having a H:C ratio close to 2 (1.73 for cellulose) while for proteins, lipids and other biomolecules this value is much lower (1.2 for Lignin, Fig. 1). In terms of processes covered in the Van Krevelen diagram (Supplementary Fig. 3), the main crowd of points is changed by dehydration, the removal of water, rather than decarboxylation which results in a change of surface charge. Decarboxylation processes being predominantly observed for peat soil under herbaceous vegetation (grasses, crops), although for only a small group of points.


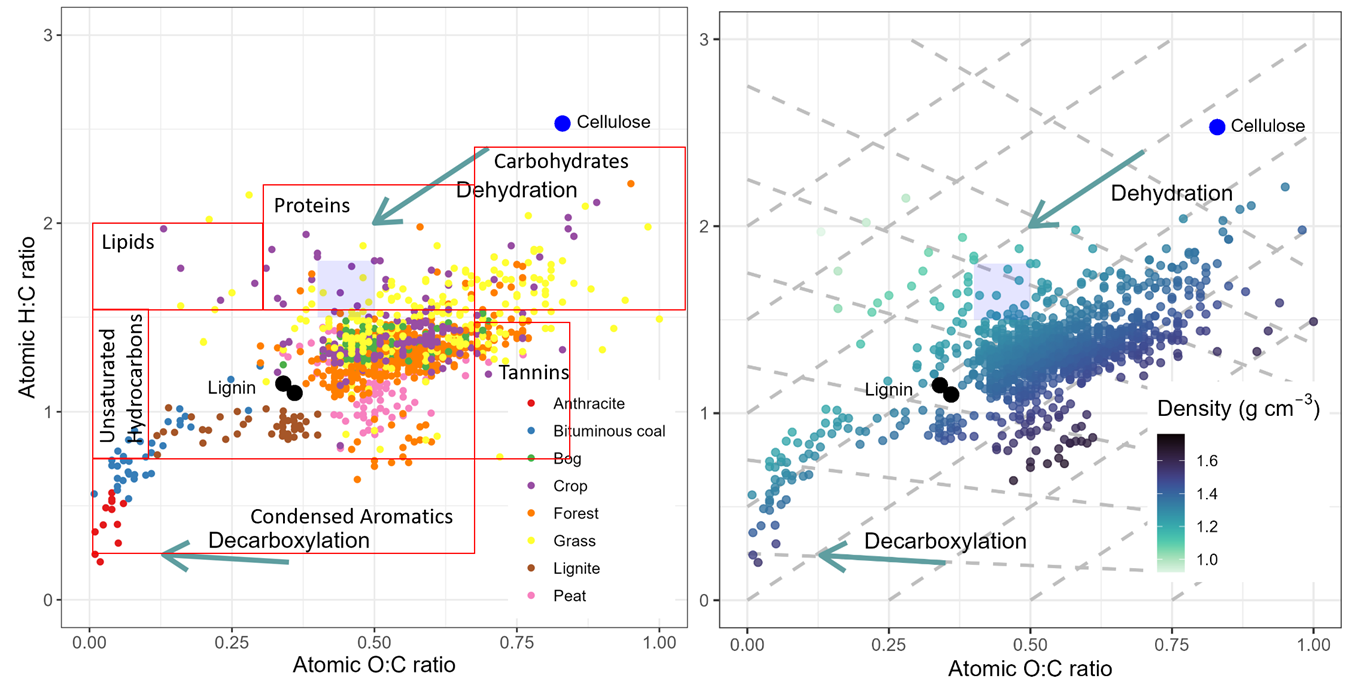


**Supplementary Fig. 3** Van Krevelen diagram of atomic oxygen to carbon (O:C) ratio and atomic hydrogen to carbon (H:C) ratios for left: coal and different land uses on peat from data by Leifeld et al. (2020) and fuel and chemical data from (Ahmad & Subawi, 2013). Lignin and cellulose are reference points for decarboxylation and dehydration processes. Overlain are the interpretive compounds associated with the respective ratios from Hockaday et al. (2009). Right: Organic matter particle density calculated by using the atomic H:C and O:C ratios displayed on the left to determine the organic matter density based on the conversion factor presented by Kuwata et al. (2012). The particle density (ρ_org_^)^ is calculated as follows the formula = [(12 + 1(H:C) + 16(O:C)] / [7.0 + 5.0(H:C) + 4.15(O:C)] valid for 0.75 < ρ_org_ < 1.90 g cm^−3^. Calculated soil organic matter density data and H:C ratios are used in Fig. 5 in the main text.


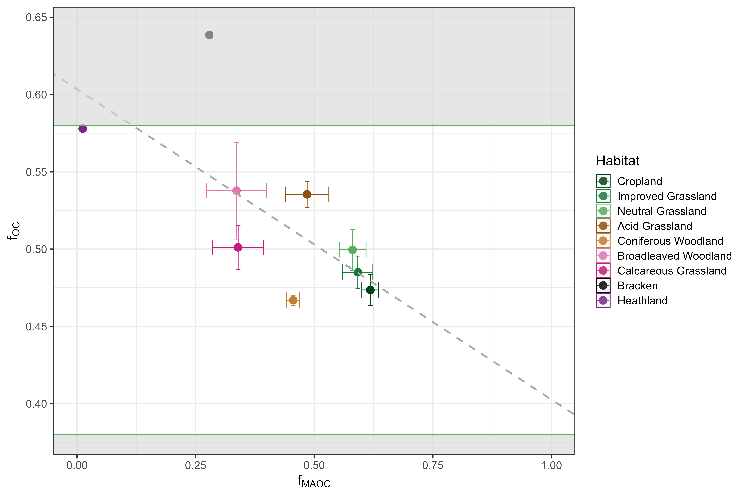

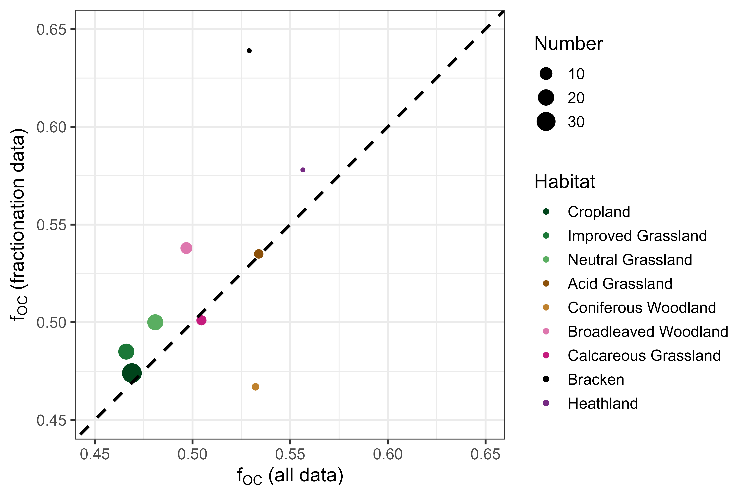


**Supplementary Fig. 4** Left: The fraction of organic carbon in soil organic matter, $f_{OC}$, correlated with fractions of mineral associated organic matter carbon in SOC (*r(90)* = -0.54, *p*<0.001), $f_{MAOC}$, across habitats. Horizontal lines show $f_{OC}$ of 0.38 and 0.58 representing the average carbon content of crop roots (38%) and humic acid (58%), respectively. The dashed grey line shows the fit to the raw data points (not shown). The dataset is biased in numbers, with highest sample numbers for Cropland, Improved and Neutral grasslands, and lowest numbers for Coniferous woodland, Bracken and Heathland (Supplementary Table 3). This figure is the counterpart to Fig. 6 of the main paper. Right: Fraction of organic carbon in soil organic matter ($f_{OC}$) across habitats for all data (Supplementary Table 2) compared to the fractionation dataset (Supplementary Table 3). Size of the points correspond to the number of samples within each habitat of the fractionation dataset (n=92) (Lebron et al., 2025). The figure shows where $f_{OC}$ of the fractionation dataset corresponds well with $f_{OC}$ of the complete dataset; that is when points are on, or close to the dashed 1:1 line. Where points are far off the line, representation of $f_{OC}$ in the fractionation dataset is not ideal for that habitat (e.g. Coniferous woodland and Bracken).

**Soil organic matter (as Loss-on-ignition; LOI) determination using different combustion temperatures**


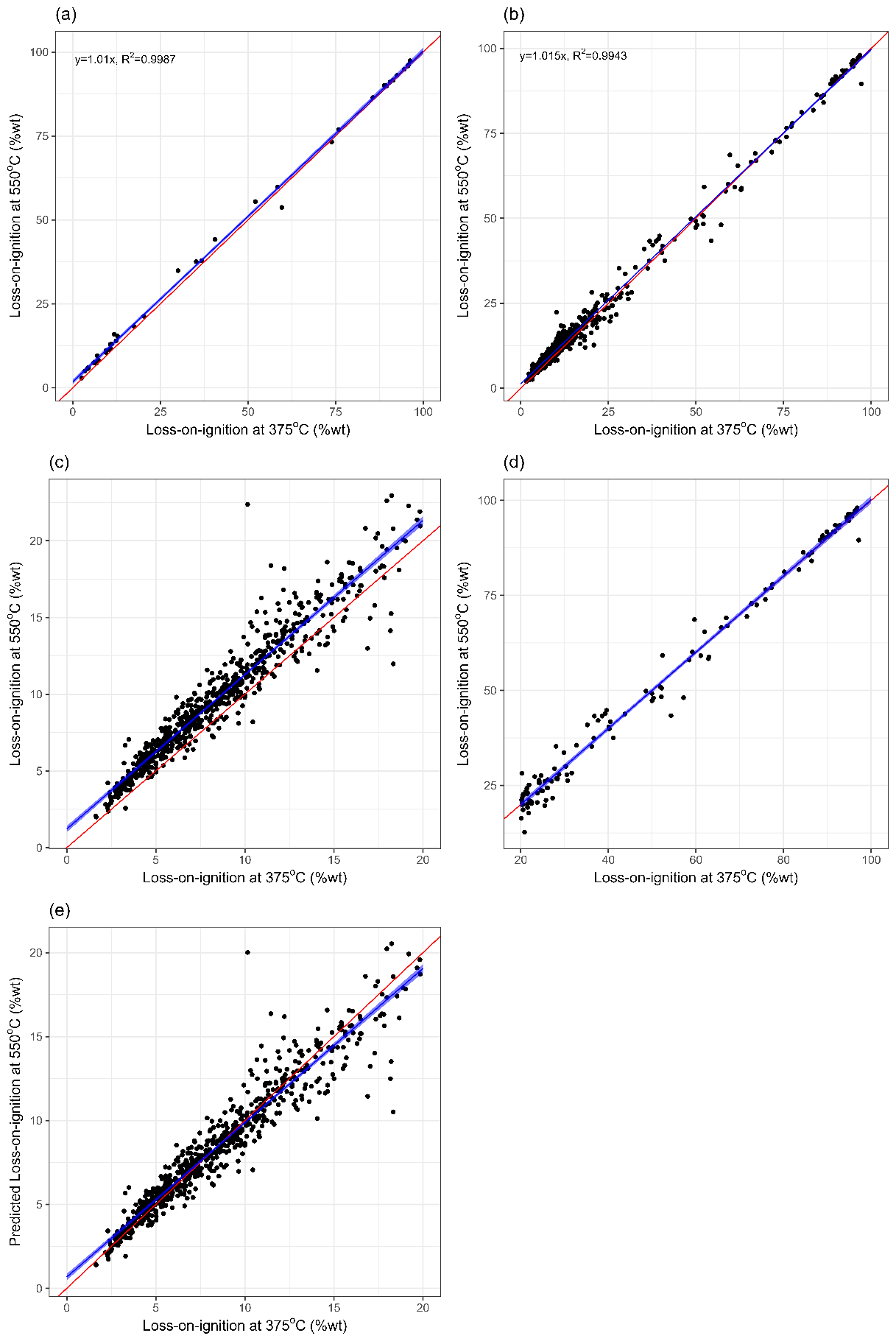


**Supplementary Fig. 5** Comparison of Loss-on-ignition measurements performed on 10 g soil at 375^o^C and 1 g soil at 550^o^C; a): data from (Emmett et al., 2010) where 40 samples were re-analysed from the same archived soil sample in 2007, b): a total of 1106 soil samples analysed for LOI on 1 g soil at 550^o^C in 1998 and re-analysed from archived soil on 10 g soil at 375^o^C in 2008, The full dataset shown in b) was divided into mineral soils defined as LOI <= 20% (c) and organic soils defined as LOI > 20% (d). e) shows the adjusted LOI values at 550oC using the relationship from c) (LOI550_predicted = 0.9141*LOI550-0.4349). The red line shows the 1:1 line, linear prediction is shown in blue including the confidence interval (light blue), with the equation plotted in each panel.


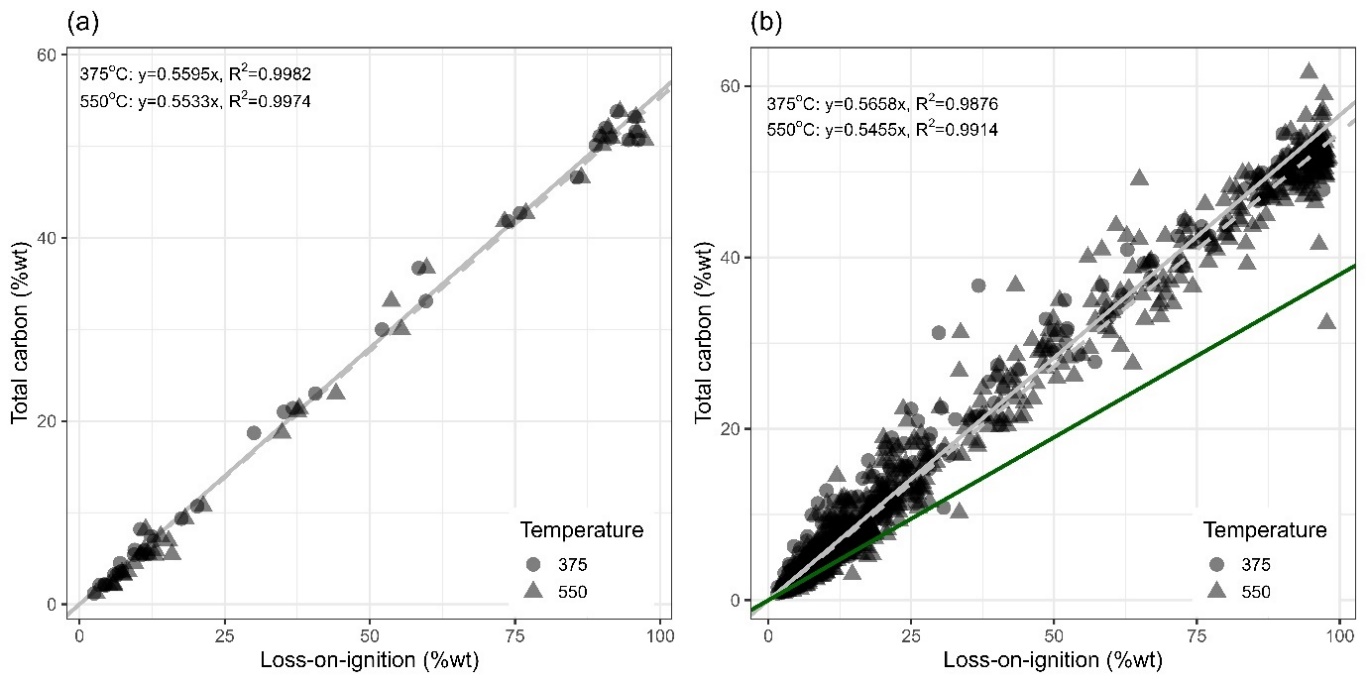


**Supplementary Fig. 6** Relationship between total carbon and soil organic matter (measured as Loss-on-ignition) at 375^o^C and 550^o^C respectively, in left: 40 soil samples (Emmett et al., 2010), and right: 1106 soil samples from Countryside Survey 1998. Linear regressions were forced through zero, with the regression plotted for both temperatures in each panel. Solid and dashed regression lines for LOI measured at 375^o^C and 550^o^C, respectively. Green line in the left panel shows the slope of 0.38 related to un-decomposed plant material.


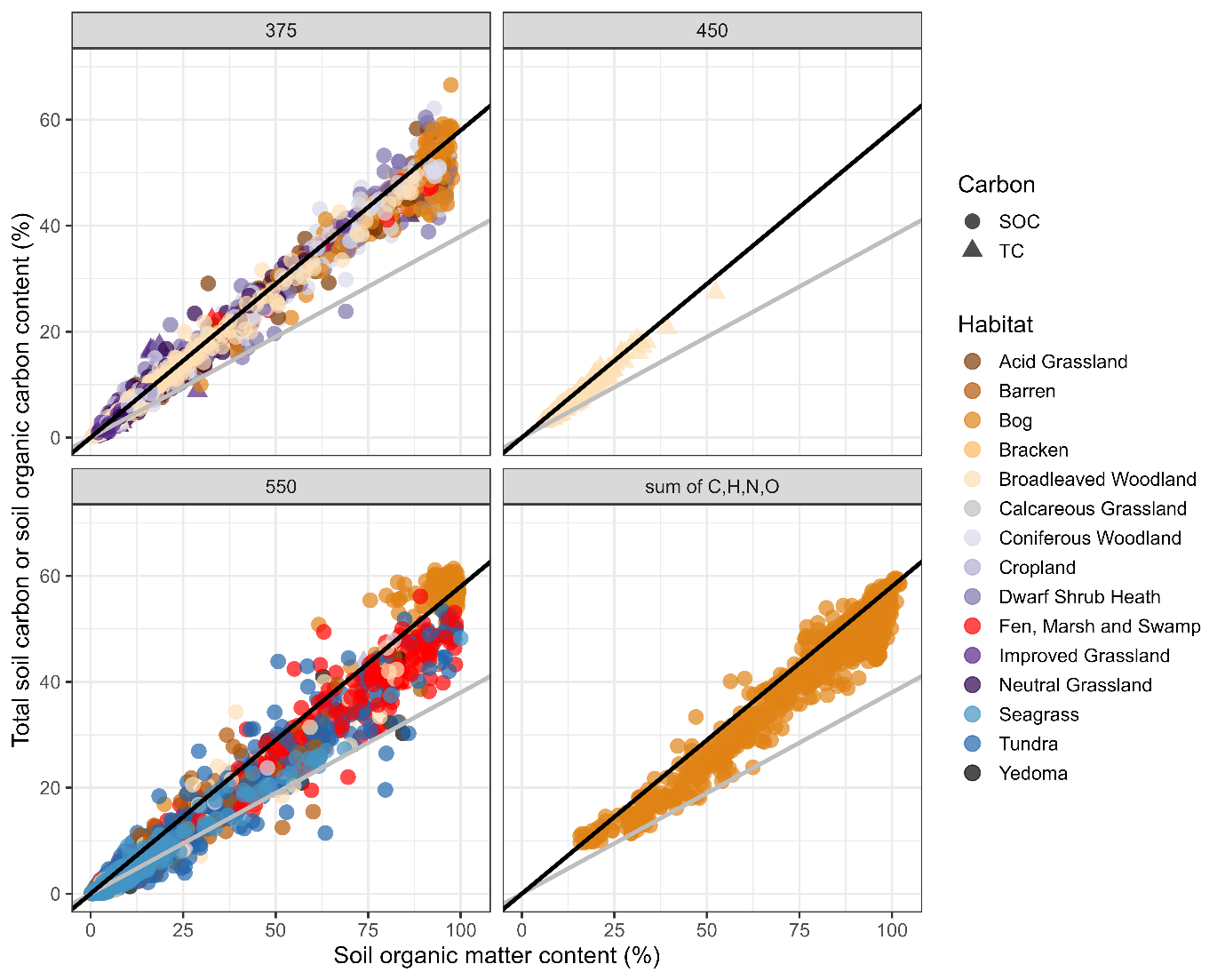


**Supplementary Fig. 7** Soil organic matter content (SOM) plotted against total soil carbon (TC) or soil organic carbon (SOC) contents for different LOI temperatures or other methods. Colours show habitats. The variation in the data at 550^o^C may be due to habitat, colder climates in the Northern permafrost regions Supplementary Fig. 8, or the structural water loss from clays at this temperature (Rich & Kunze, 1964). The LOI method may have an effect on SOM estimates; however, this has not been included in the statistical analysis as a) habitats captured in the 375^o^C and 550^o^C data subsets are considerably different, and b) that our independent dataset where soil samples were measured for LOI at 375^o^C and 550^o^C on the same sample (Supplementary Figs. 5 and 6) did not show this discrepancy to that degree.


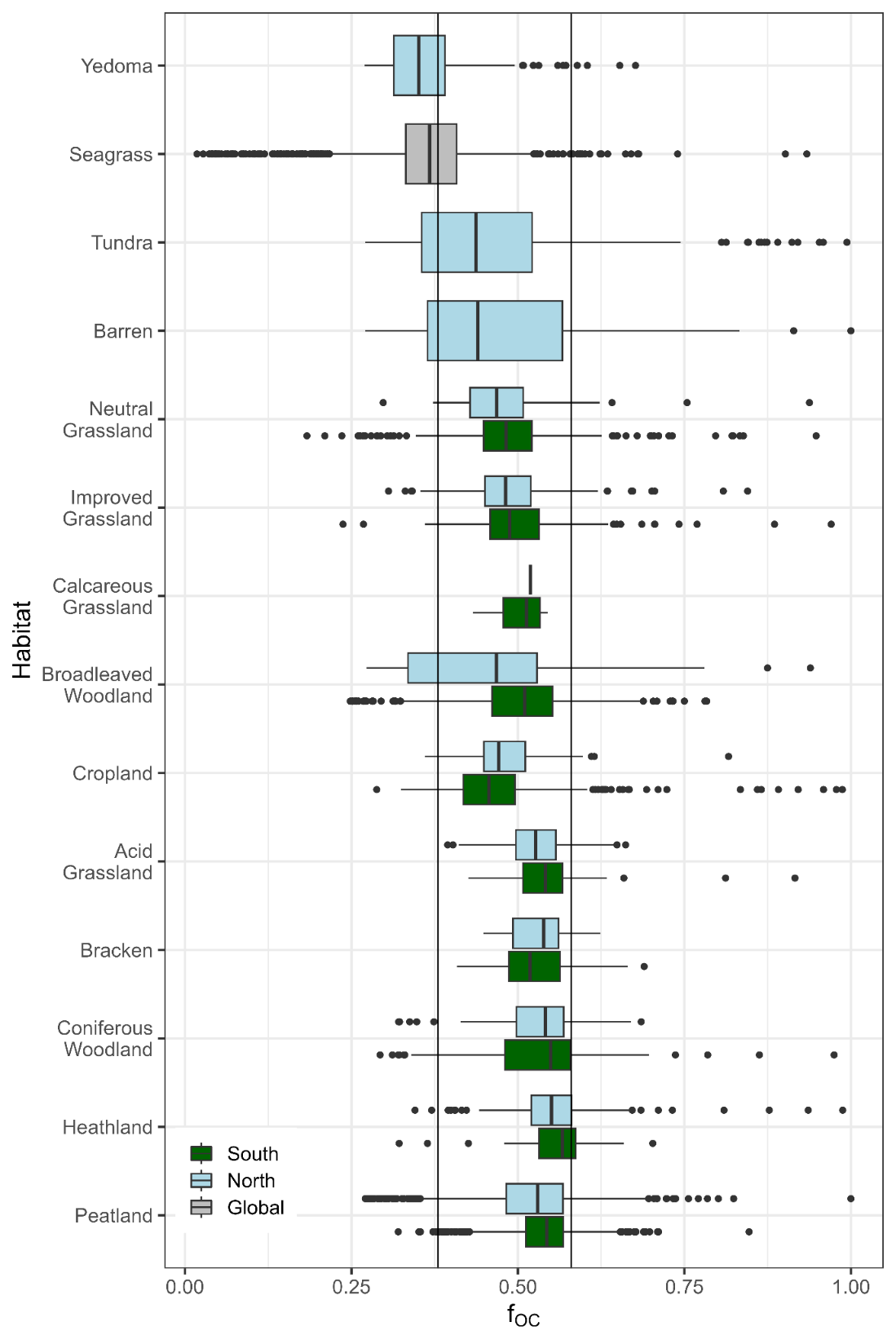


**Supplementary Fig. 8** Fraction of organic carbon in soil organic matter ($f_{OC}$) across habitats for Permafrost region and Northern European countries (Blue: Canada, Finland, Greenland, Norway, Russia, Scotland, Sweden), Southern European countries (green: England, Spain, Switzerland, Wales), and grey: Global (J. W. Fourqurean et al., 2012). The two black lines correspond to the average carbon contents in crop roots (38%) and humic acid (58%) in line with Fig. 1.


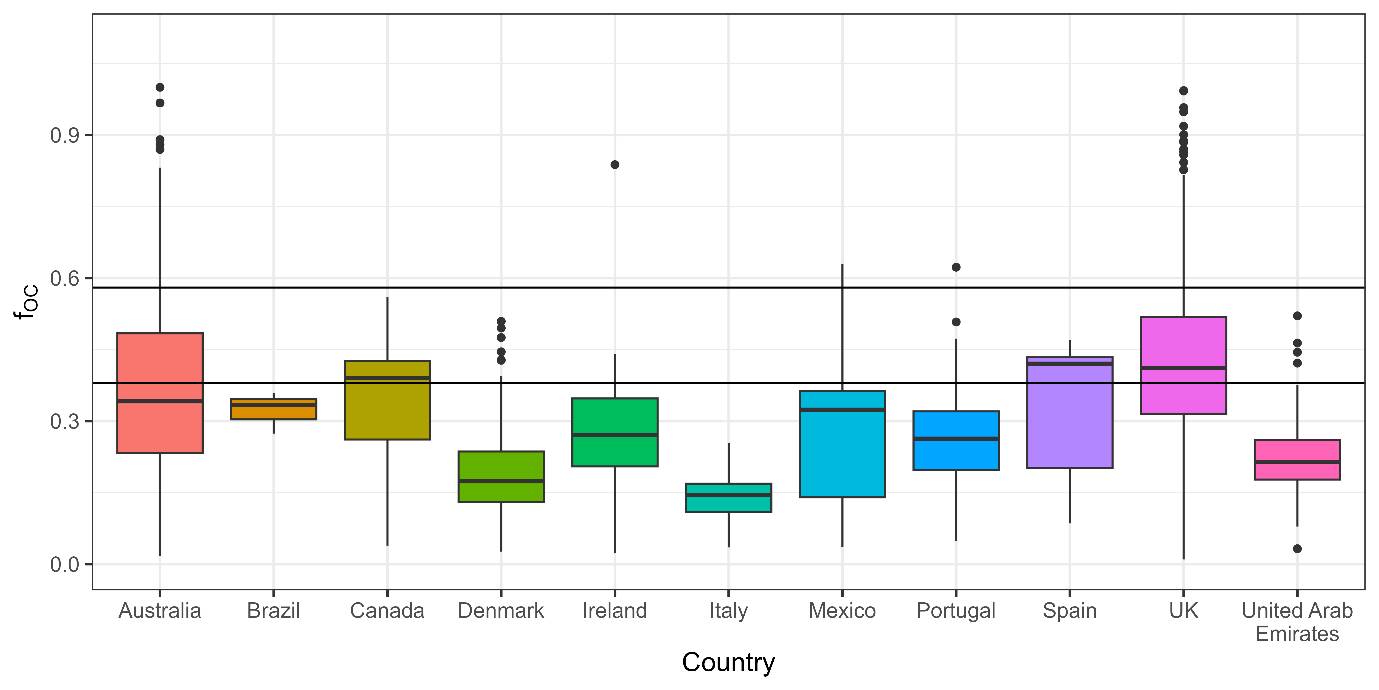


**Supplementary Fig. 9** Fraction of organic carbon in soil organic matter ($f_{OC}$) calculated for the MarSOC dataset on tidal marshes by Maxwell et al. (2023). Data points were included where SOC and SOM measurements were marked as observed and occurred on the same sample. Data were plotted as provided. Mean $f_{OC}$ across the 1441 data points is 0.33, median $f_{OC}$ is slightly lower (0.31). Data were not included in the analysis in the main text as high $f_{OC}$values, especially in Australia and the UK datasets could not be quality assessed without reviewing individual datasets.

**Sampling and analyses methods used across datasets**

**UK National-scale datasets**

The Countryside Survey is a national integrated survey of the United Kingdom (UK, (Keith et al., 2020; Reynolds et al., 2013)). As part of the Countryside Survey, topsoil (0-15 cm) was first sampled in 1978, then re-sampled in an expanded survey in 1998, 2007 (Emmett et al., 2010; Reynolds et al., 2013; Robinson et al., 2024), and the most recent rolling survey covering 2019-2023. The Countryside Survey features a stratified random sampling, designed to detect state and change (Robinson et al., 2024). UKCEH Countryside Survey topsoil samples from 2018-2023 (Bentley, Brentegani, et al., 2024; Bentley, Reinsch, Alison, Andrews, et al., 2023; Bentley, Reinsch, Alison, Brentegani, et al., 2023; Bentley, Reinsch, Brentegani, et al., 2023; Reinsch, Bentley, et al., 2023) and winter 2021(Bentley, Reinsch, et al., 2024), were analysed. Soils were dried at room temperature and sieved to 2 mm prior to SOC and SOM analyses. Samples were analysed on a TGA (TGA 8000, PerkinElmer. IR: PerkinElmer Spectrum Two), where data on Loss on Ignition (LOI) at 375^o^C and CaCO_3_ contents were extracted (Lebron et al., 2024). Total carbon was measured using an Elementar Vario-ELelemental analyser (Elementaranalysensysteme GmbH, Hanau, Germany) following the UKAS accredited method SOP3102, at UKCEH Lancaster. Information on land use was extracted from the associated botanical datasets. Data points on urban soils, inland rock, boundary and linear features, littoral and supra-littoral sediments were removed from the datasets as these are very specific habitats with only a small number of data points in each category.

The Environment and Rural Affairs Monitoring & Modelling Programme is an ecological survey of the Welsh landscape between 2021 and 2025 (Reinsch, Bentley, et al., 2025). Topsoil (0-15 cm) collected during this re-survey and were analysed in the same way as soils from the Countryside Survey described above. Land use was determined by a co-located vegetation survey in the same way as for the Countryside Survey (Wood et al., 2021).

**Northern Permafrost region**

A soils dataset was collected for the northern permafrost region which can be used to describe spatial patterns in soil properties (Palmtag, Obu, Kuhry, Richter, et al., 2022; Palmtag, Obu, Kuhry, Siewert, et al., 2022). A total of 16 sampling sites with 11 sites in permafrost regions across five countries (Sweden, Norway, Russia, Canada, Greenland) in the Northern Hemisphere were sampled. Samples were heated to 105^o^C with LOI being determined at 550^o^C. If LOI at 950^o^C following Heiri et al. (2001) indicated presence of inorganic carbon above 1%, samples were acid treated with hydrochloric acid prior to determination of total carbon on an elemental analyzer (EA). Land cover was derived from satellite products, classed into primary land use classes (forest, tundra, wetland, barren, Yedoma sediment). Tundra comprised shrub tundra and graminoid/forb tundra. Soil samples were taken between 2006 and 2019, usually during the summer months. The sampling frequency was predominantly determined by Pedon using representative landscape units and types, maps, and remote sensing products prior to field work. For each Pedon, the organic layer and the active layers were sampled from an open soil pit excavated to the bottom of the active layer to the bedrock, or to a depth of at least 50 cm. Deeper unfrozen soil layers were sampled using a steel pipe. Samples of the active layer were sliced into 5 cm intervals. Blank CaCO_3_ cells were filled with zero reasoning that no CaCO_3_ was expected in these soils and thus were not analysed for contents.

**Peatlands**

Five upland ombrotrophic blanket bogs were sampled across England (n=2), Scotland (n=2) and Wales (n=1) (Toberman et al., 2016). All data points were classified as Bog. Triplicate adjacent cores were extracted in 2014 using both a box corer (to recover the surface vegetation and uppermost 1 m of peat) and a Russian-type corer (for peat deeper than 1 m), which were sliced in 10 cm intervals. Soil samples were analysed for LOI (550^o^C) and total carbon was measured after drying at 105^o^C on an Elementar Vario-EL analyser (ISO17025). Total carbon in bogs corresponds to SOC.

Data from the UK datasets classed as Fen, Marsh and Swamp were grouped together in the peatland category lowland peats (such as Fen Marsh and Swamps) are considered limited by higher water content and lower oxygen availability.

Data were collected in a recent study looking into peat degradation with land use in Switzerland (Leifeld et al., 2020). A total of 48 peat sites with four land uses, including cropland, forest, grassland, natural peat, were sampled. For this study, data were classified as Cropland, Coniferous Woodland (assuming that most forest on Peat would fall in this category without more information presented in Leifeld et al. (2020)), Improved Grasslands (assuming that most grasslands if drained would be used as this), and natural peat (classed as Peatland in this study). Soil samples were taken in triplicate per site 25 m to 50 m apart. Cores were cut into 3 cm to 10 cm increments, dried at 105°C, milled, and measured for C, H, and N by dry combustion and elemental analysis, and for O after pyrolysis at 1000°C and subsequent GC-TCD quantification (Hekatech, Germany). SOM was calculated as the sum of the four elements. A subset of samples was fumigated with HCl before elemental analysis; these soils did not contain carbonates.

**Seagrass and Saltmarshes**

A global database of seagrass sediment organic carbon stocks was assembled to assess the impact Seagrass ecosystems have in the global carbon budget (Fourqurean, 2012; Fourqurean et al., 2012). The database contains a total number of 3561 measurements, of which 1384 data points contained both SOC and SOM measurements and passed the QA for this paper.

Saltmarsh data were collated for a global dataset (MarSOC) on tidal marshes (Maxwell et al., 2023). Data were collected from 99 tidal marsh peer-reviewed and unpublished studies featuring a total of 17454 data points. Of those, 1441 data points had SOC and SOM measurements which originated from 11 countries from across the world (Supplementary Fig. 9). The MarSOC dataset was quality checked as detailed in (Maxwell et al., 2023). Data were not included in the analysis in the main text as high $f_{OC}$ values, especially in Australia and the UK datasets could not be quality assessed without reviewing individual datasets.

**Woodlands**

Forest Research BioSoil forms a large EU forest soil and biodiversity survey, which was undertaken during 2005–2009 (Vanguelova et al., 2013). Sites were selected on the basis of the presence of woodland on a 16 x 16 km national grid and covered a wide range of tree species and soil types. In Great Britain, of the total of 220 BioSoil plots, 76 are in England, 32 in Wales and 112 in Scotland (Reinsch, 2025). One representative soil pit and five randomly sampling points were located within each circular plot (25 m radius), and the soil sampled incrementally down to 80 cm (0–5, 5–10, 10–20, 20–40 and 40–80 cm soil depths; soil horizon samples taken from the soil pit; (0 cm taken as the top of the mineral soil) and top of the peat horizon in peat/peaty soils using a Dutch soil auger. Sampling and analysis of biomass and C content of litter (L) and fermenting humus (F) and peat (H) horizons were taken in triplicate per plot. Soil sampling and analyses were carried out according to the UNECE ICP Forest Manual for Soil Sampling and Analysis (Cools & De Vos, 2020). Measurements and analyses performed on BioSoil samples include soil organic and inorganic C concentration for each soil horizon (L, F, H, A, B and C) and five soil depths (0–5, 5–10, 10–20, 20–40 and 40–80 cm). Chemical analyses were performed by the Forest Research chemical laboratory at Alice Holt, and quality was assured by rigorous EU interlaboratory comparison. SOC was analysed by dry combustion at 900°C using a C/N analyser (CE Instruments Ltd, FlashEA 1112 Series). LOI was determined at 375^o^C for most of the English BioSoil plots.

A UK study was conducted to identify substitute host tree species for epiphytes (Mitchell et al., 2021). Co-variates collected were SOC and SOM and are available from Mitchell et al. (2021). Soil samples were taken from six sites across the UK that were previously old country houses with large formal gardens and parklands. Historical management allowed for the selection of old, often more than 150-year-old, broadleaved trees. Information on tree location is available (semi-natural woodland, grassland-park, or garden). Semi-natural woodland was classified as Broadleaved woodland, whereas grasslands and gardens were assigned to the Improved grassland category. There were two sites in England, Wales, and Scotland, respectively. A total of eight soil samples were taken per tree (diameter 25 mm, depth 10 cm) about 2 m from the tree trunk and evenly spaced around the tree. Soil samples were bulked per tree and kept cool until they were processed. LOI was determined at 450^o^C (Mitchell et al., 2021). A sub-sample of the 2 mm sieved soil was ball milled (MM22 Mixer Mill, Retsch) and analysed for total carbon following the method in Pella & Colombo (1973).

Another UK dataset was collated on woodland-grassland contrasts to study the impact of land-use on SOC dynamics to depth (Reinsch et al., 2024). Five locations were sampled across England featuring different soil types but similar land uses. Woodlands were paired with bogs or neutral grassland vegetation. Soil samples were taken in a randomized grid, featuring nine soil samples to 1 m depth for each land use per location. Samples were analysed for LOI, total carbon and SOC using the methods described for the UKCEH Countryside Survey described above.

**Grasslands**

A dataset from across the UK was collated (Reinsch, 2025) as part of the UK-China Virtual Joint Centre for Improved Nitrogen Agronomy which aimed, amongst others, to develop novel indicators of soil quality. Topsoil (0-15 cm) samples were initially dried at 25^o^C for 14 days. LOI was determined on a Carbolite furnace at 375ºC. Total carbon was measured using an Elementar Vario-ELelemental analyser (Elementaranalysensysteme GmbH, Hanau, Germany) following the UKAS accredited method SOP3102, at UKCEH Lancaster. Ten locations were visited across the UK with the aim to cover as many managed and (semi-) natural land uses as possible. Data are available for Acid, Calcareous, Improved and Neutral grasslands, as well as for Cropland, and Fen, Marsh and Swamp.

Soil datasets were collected in Greenland between 2015 and 2018 to study soil water repellency, particle density, and soil water retention in sub-arctic pasture and grass fields under global change(Weber et al., 2021, 2022, 2023). The study sites cover the main agricultural region of Southern Greenland, which is absent of permafrost. Air-dry soil was added to clean and pre-ignited crucibles, and oven-dry soil weight was determined after oven-drying at 105◦C for 24 h in a ventilated oven. The crucibles were subsequently stepwise ignited at 225◦C and 550◦C for 12 h in a half-full Nabertherm L 40/11 muffle furnace (Nabertherm GmbH, Lilienthal, NI, Germany) to ensure an even ignition. None of the soils tested positive for calcium carbonate (verified by adding 10% HCl). Therefore, the organic carbon could be set equal to the total carbon obtained from dry combustion using an ELTRA Helios C-Analyzer (ELTRA GmbH). The agricultural land use was pasture, cultivation and extensive cultivation which we grouped into either the Improved or Neutral grassland categories.

A set of Spanish soils featured mainly Improved and Neutral grasslands, but also Coniferous woodland (Reinsch, 2025). Four sites were sampled for four different land uses from the topsoil 0-20 cm. Each land use was at least sampled in three replicates per land use. SOM was determined using the method used for the UKCEH Countryside Survey samples described above. Total carbon was determined using dry combustion (elementary analysis, ISO 10694:1995) and was corrected for CaCO_3_ content (Supplementary Table 1, Supplementary Fig. 10).

**Croplands**

The Finnish national soil monitoring network for Cropland was established in 1974, with resampling conducted in 1987, 1998, 2009, and 2019 to assess the chemical status of the soil (Soinne et al., 2022). The data has also been utilized to explore the potential of agricultural soils to act as carbon sinks for atmospheric carbon capture (Heikkinen et al., 2022). This study was based on the subset of new 150 sampling plots introduced in the network in 2018 when SOM and total carbon were measured (Heikkinen et al., 2021). Soils were sampled to 40 cm soil depth (4.8 cm diameter). In each plot, three replicate soil samples were taken three metres from the plot centre. The soil was sieved (2 mm), then dried to constant weight at 105^o^C and thereafter ashed at 550^o^C until at constant weight using a Leco TGA 701 (LECO Corporation, MI). Total carbon of the sample was analysed by dry combustion (Leco TruMac CN, LECO Corporation, MI) at the soil laboratory of the Natural Resources Institute Finland.

An unpublished dataset featuring Croatian soils from seven sites featuring Cropland and Orchards, of which Orchards are categorised as Broadleaved woodland. Soil samples were taken in 25 cm depth intervals to one metre depth. LOI and total carbon were determined using the methods described for the UKCEH Countryside Survey soil samples. The Croatian dataset was not included in the final data analysis because we assumed that not all carbonates were calcium carbonate, and thus, SOC could not be calculated reliably using CaCO_3_ correction as indicated by $f_{OC}$ for the habitats present in the dataset (Supplementary Fig. 10).

**Laboratory methodology considerations**

Methodologically, SOM is usually measured using loss on ignition (LOI). Given that different temperatures are used this could affect the comparability of the aggregated data (Pribyl, 2010). Data collection was constrained to data sets produced using well documented methods at 375^o^C, 450^o^C or 550^o^C, and for peatlands the sum of the elements C, N, O, and H (Supplementary Table 1, Supplementary Fig. 7). Moreover, by examining an independent, national dataset (n=1106) it showed that SOM measured as LOI at 375^o^C (16 h) or 550^o^C (4 h) on the same samples follows a 1:1 line with a slope of 1.015 (*R*^2^=0.9943) (Supplementary Fig. 1). Looking at the fraction of total carbon in SOM for both, 375^o^C and 550^o^C, with slopes of 0.57 and 0.55 (Supplementary Fig. 6), points scatter considerably, leading to the conclusion that although a higher LOI temperature slightly over-estimated SOM content, this is negligible when combined with the uncertainty in SOC determination in the case of this data set. We acknowledge that measurements on soils containing substantial quantities of clay minerals, with crystal water, may not conform as water is released at the higher temperatures (>450^o^C) (Rich & Kunze, 1964). The deviation of data points from expected SOC values for lower SOM contents is driven by inorganic carbon such as calcium carbonate as measured by total carbon (Supplementary Fig. 10), and as well-known, total carbon values need to be corrected for inorganic carbon to report SOC. If inorganic carbon was not removed from the soil samples by HCl (usually 10%) prior to total carbon determination, the correction for inorganic carbon was done assuming all the inorganic C in the soil is CaCO_3_. However, presence of other carbonates, like dolomite or Na_2_CO_3_ makes the correction challenging and is no substitute for the elimination of carbonates prior to total carbon analysis.


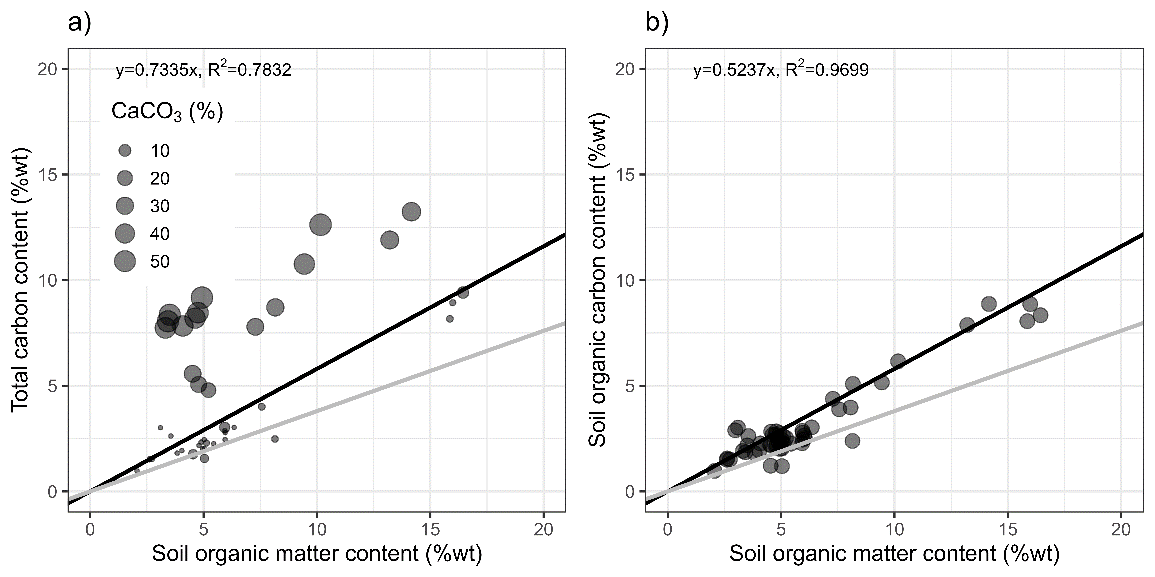


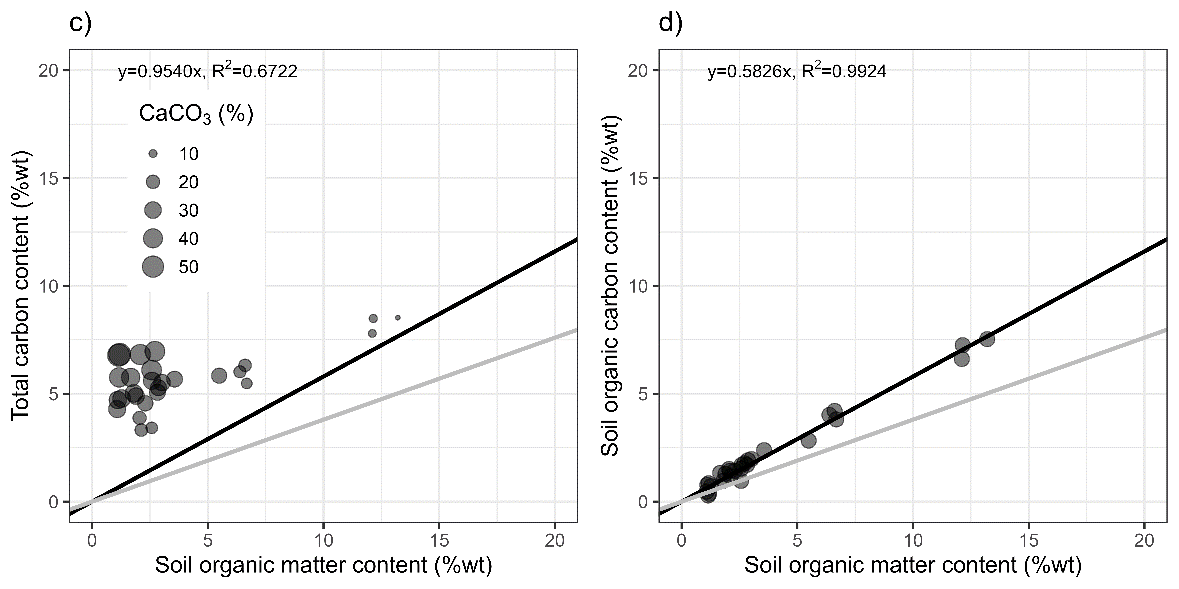


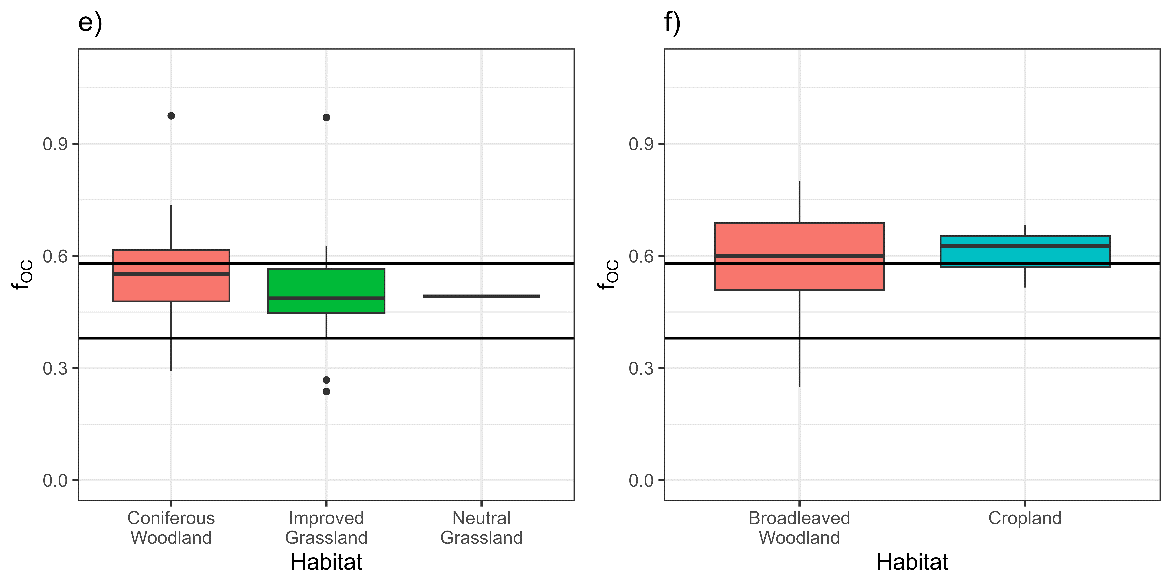


**Supplementary Fig. 10** Relationship between soil organic matter and a+c) total soil carbon and b+d) total soil carbon content corrected for CaCO_3_ (=SOC), and e+f) habitat for a+b+e) 55 Spanish soil samples, and c+d+f) 28 Croatian soil samples. SOM was measured for both soil datasets using the Loss on ignition method as the difference in weight of a sample between 105^o^C and 375^o^C for 16 h.

**Methodological considerations**

While data analysis showed that measurements at 375^o^C and 550^o^C are comparable due to the difference in time, combustion at lower temperatures for SOM detection is preferred (Lebron et al., 2024). This is because, although the Countryside Survey samples analysed had low amounts of clay minerals, clay minerals release structural clay mineral water from about 550^o^C (Rich & Kunze, 1964), resulting in an overestimation of SOM. The temperature used for determining SOM from LOI should be, as already suggested in Pribyl (2010), and further evidenced by Lebron, Cooper, et al. (2024), below 450^o^C to avoid the burn-off of black C. Thermogravimetric analysis (TGA) offers an important tool for cost-effective analysis to help understand a wide range of soil including soils with black carbon and carbonates (Lebron et al., 2024).

TGA can also inform of the presence of inorganic carbon. If the form of carbonate in the soil is known, TGA data and the stoichiometry of the carbonate can be used to subtract inorganic carbon from total carbon to derive SOC. However, this approach is not possible if mixtures of carbonates are present in the soil, or if the type of carbonate is unknown. In such cases, carbonates need to be fully removed before total carbon analysis to derive SOC.

If reported SOC still contains inorganic C due to incomplete removal, this will lead to overestimation of SOC stocks, and erroneous use of the relationship between SOC and SOM. Looking at the distribution of pH ranges across Europe using the LUCAS dataset, Lu et al. (2023) mapped pH across the continent, highlighting areas of pH > 7.6 when carbonate formation is possible. Zamanian et al. (2024) estimated that 93.5% of cropland soils across Europe contain carbonates, of which 74% and 36% contain carbonates above 1% and 5%, respectively (Even et al., 2024).

**Advantages of good SOC estimates**

Independent validation of impactful national-scale datasets is of high importance as they inform the trend in national and global SOC estimates. No laboratory is free of instrumental error (Even et al., 2024) and in recognition, reference standards, such as Acetanilide for carbon content and internal reference standard(s), should be standard procedure as well as voluntary laboratory-inter-comparisons. However, reference standards often don’t capture the wide range of measurement values. SOC and SOM (as LOI) are measured using different independent instruments and thus, are good in helping validate each other. Both, LOI for SOM and total carbon or SOC using combustion, have their limitations. What is clear is combining these measurements is much more powerful than considering each on their own.

**Microbial carbon contents**

A meta-analysis by Zhang & Elser (2017) reports on the C:N:P stoichiometry in fungi. Unfortunately, many carbon values were not available from primary studies and a value of 44% was applied to most data records. Given this lack of information, we are using reported organic carbon contents for ectomycorrhizal fungi (necromass of *Meliniomyces bicolor*) (Fernandez & Kennedy, 2018) and *Escherichia coli* (Heldal et al., 1985) as reference points for microbial organic matter content (Fig. 1).

**Soil organic matter fractionation method**

The fractionation method is detailed Lebron et al. (2025). A detailed method was slightly modified as stated in the main text: the occluded fraction was determined by mass balance given the following rationale: The process of sonication applied to the sample facilitates the breakdown of aggregates, thereby releasing the occluded fraction. This results in a significant number of colloidal-sized particles that quickly obstruct the filter during the collection and rinsing of sodium polytungstane from the occluded fraction. Consequently, this slow filtration process extends the duration of each batch by an additional two days for the organic matter fractionation separation. Therefore, based on our previous fractionation exercise where we successfully collected the occluded fraction and achieved an excellent recovery of total carbon (as reported in Reinsch et al. (2024)), we have concluded that the occluded fraction step can be eliminated from the methodology.

**Statistical approach**

The below Figures visualise the data which led to the final model structure as described in the main paper. Firstly, we confirmed a (near) normal distribution of the data (Supplementary Fig. 11), then we looked at the distribution of soil type within each habitat (Supplementary Fig. 12). Supplementary Fig. 13 explores the relationship between SOC and $f_{OC}$ which shows a general positive relationship by soil type. This interaction term was added to the final model as a consequence. The relationship between SOC and $f_{OC}$ affected by LOI method was explored in Supplementary Fig. 14. LOI method seems to change the relationship between SOC and $f_{OC}$. However, as shown in Supplementary Fig. 7, habitats are not equally distributed between methods and habitat was seen as a confounding explanatory effect in the model. Nevertheless, we tested the model by including the method term, which reduced the explanatory power. Consequently, LOI method was not included in the final model. Lastly, the factor “dataset” was considered as a random effect but excluded because, as for LOI method, habitats are not equally distributed across datasets and a confounding effect was assume, and adding this factor to the model did not improve its performance.


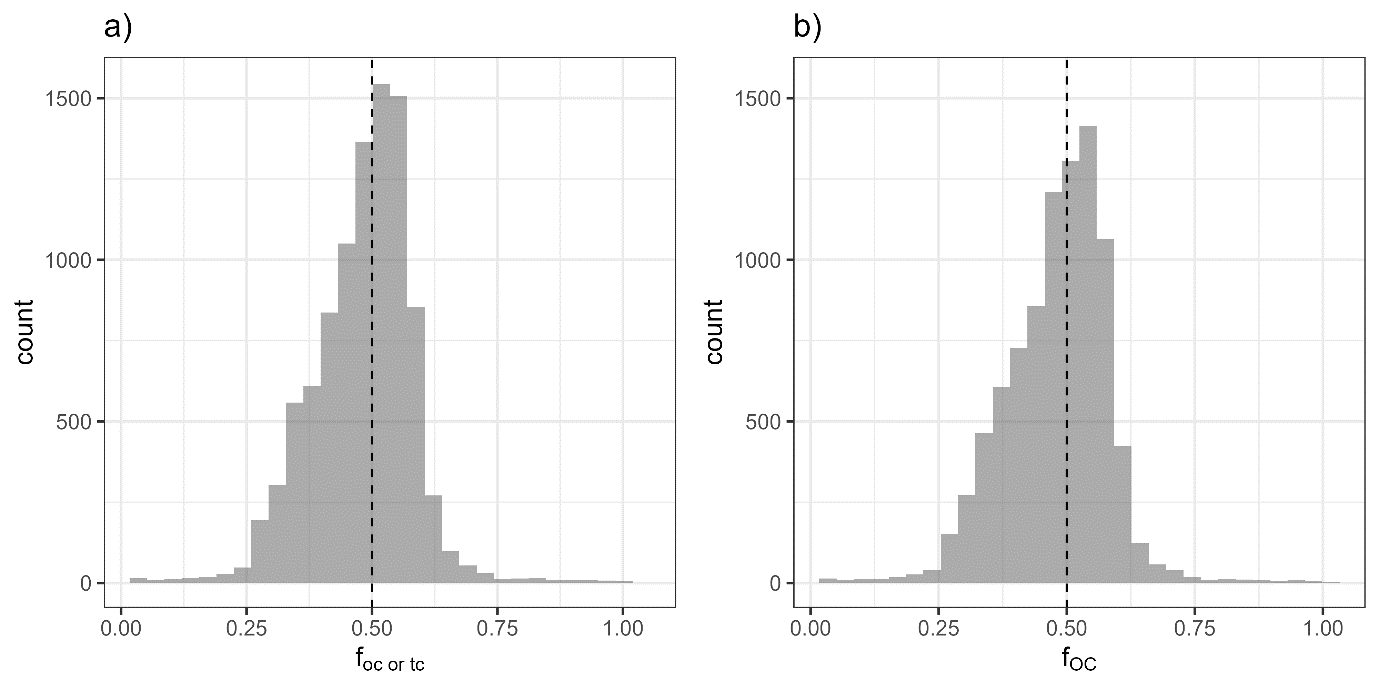


**Supplementary Fig. 11** Data distributions for the fraction of organic carbon (OC) or total carbon (TC) in soil organic matter for a) $f_{oc or tc}$ and b) $f_{OC}$.

We chose a linear hierarchical (mixed-effect) model accounting for terms identified above, allowing for different relationships with soil type (mineral and organic soils, permafrost and sediments). We also need to account for the lack of independence among some of the data: that multiple samples from the same individual cores will have their own characteristic errors.


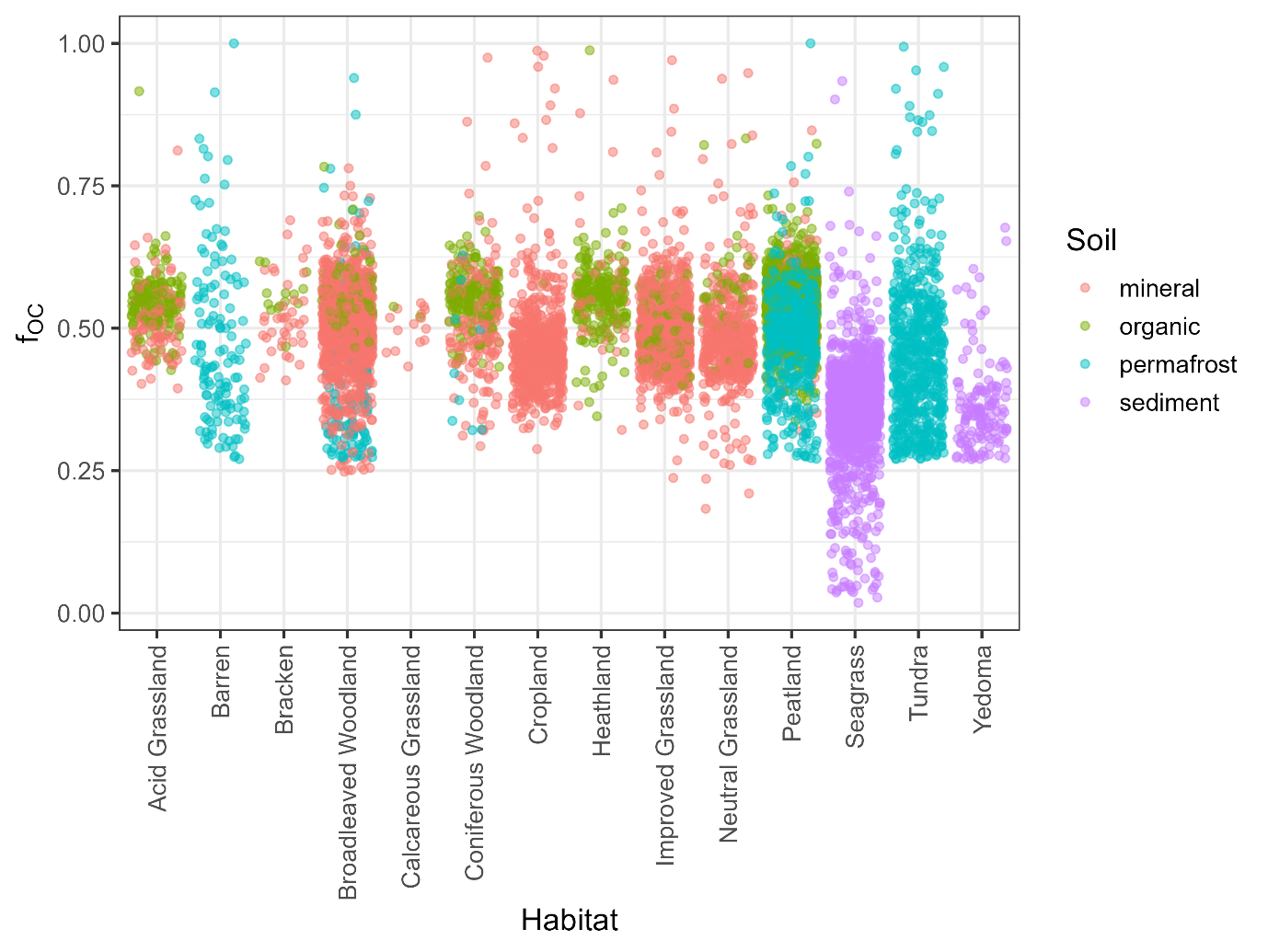


**Supplementary Fig. 12** Variability within the fraction of organic carbon (OC) in soil organic matter, $f_{OC}$, within habitats and across assigned soil types: mineral (soil organic matter (SOM) content below 20%), organic (SOM > 20%), permafrost (from permafrost regions unless Yedoma sediment), and sediment (Seagrass habitat and Yedoma sediments from permafrost regions).


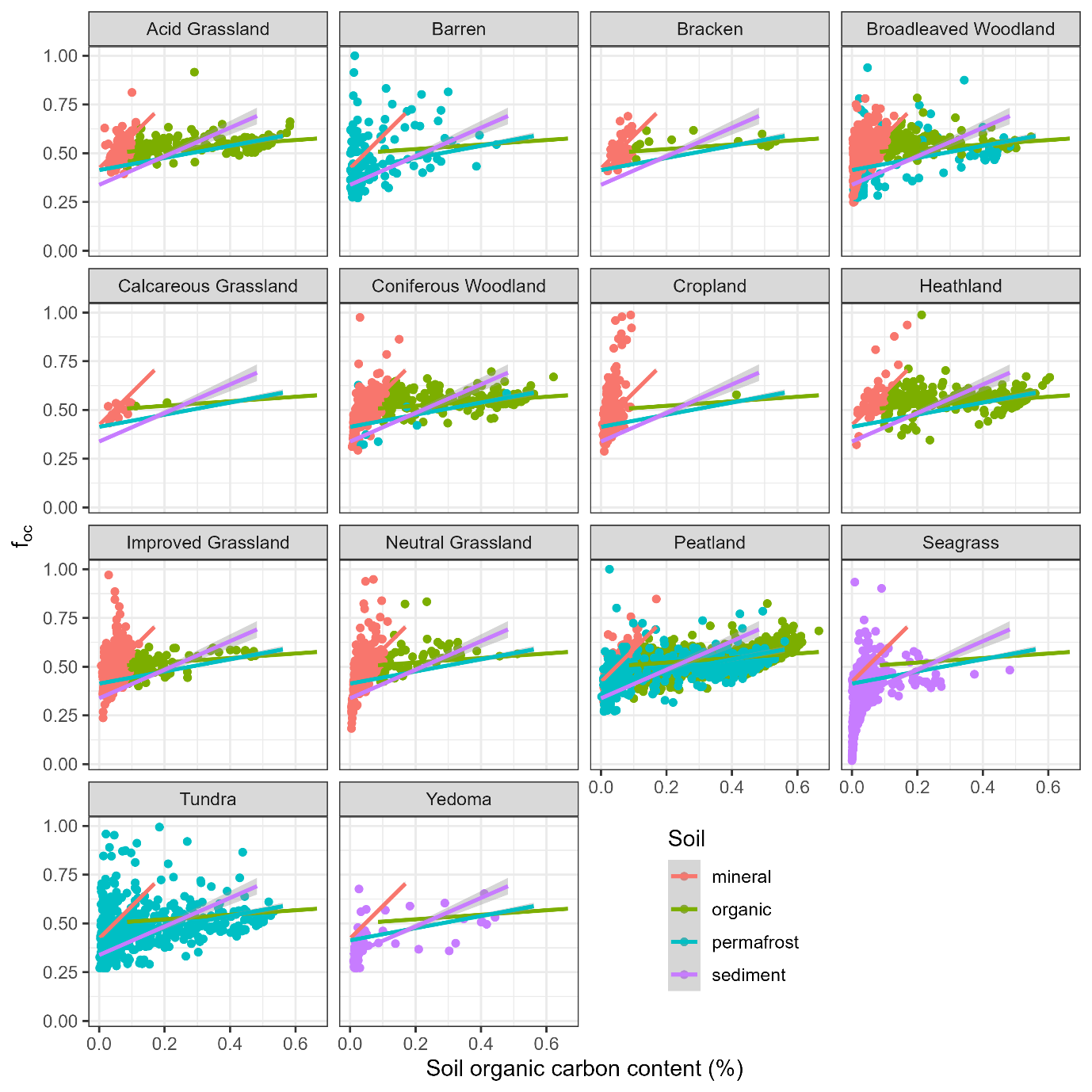


**Supplementary Fig. 13** Relationships between soil organic carbon (SOC) contents in soil and the fraction of organic carbon in soil organic matter ($f_{OC}$) for soil types within each habitat. The full dataset (including total carbon) shows the same patterns and is not repeated here.


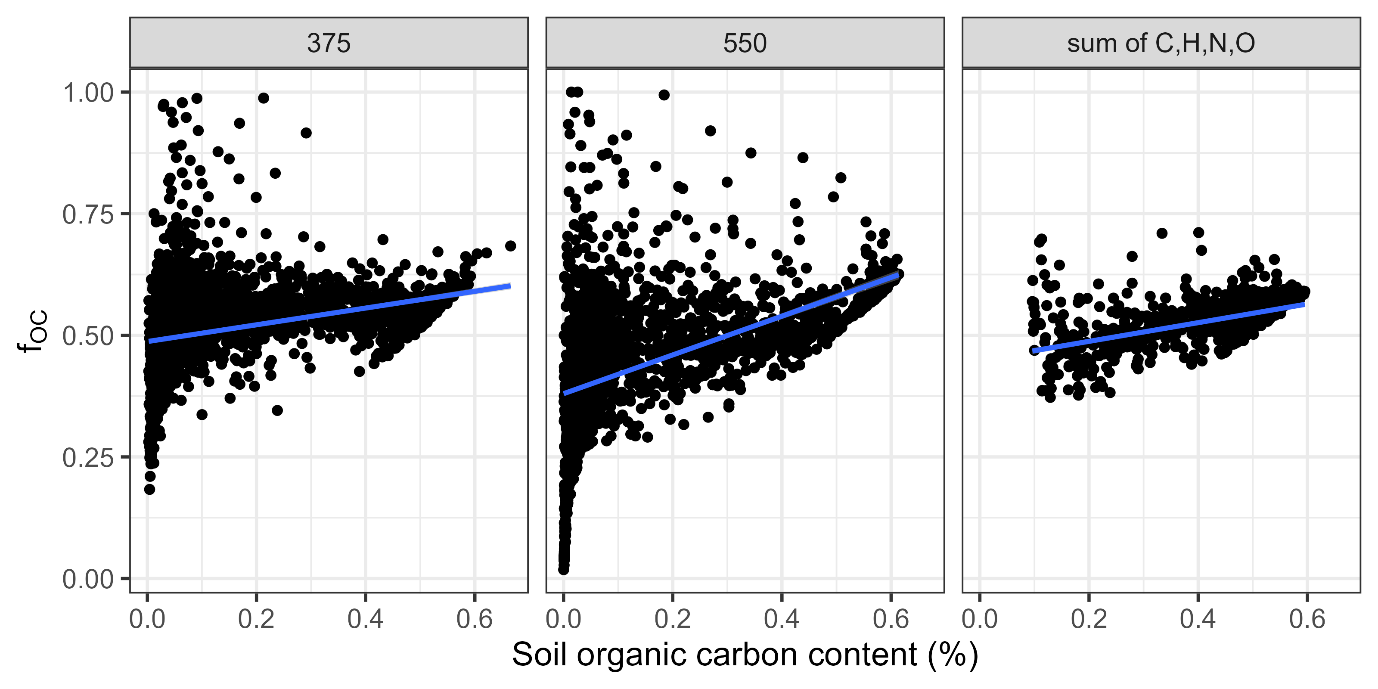


**Supplementary Fig. 14** Soil organic carbon content (%) against organic carbon (OC) in SOM ($f_{OC}$) for 8936 data points where SOM was calculated from the mass loss (usually) between 105^o^C and 375^o^C or 550^o^C. For organic soils, SOM can also be calculated from the sum of C, H, N and O. This figure also relates to Supplementary Fig. 7.


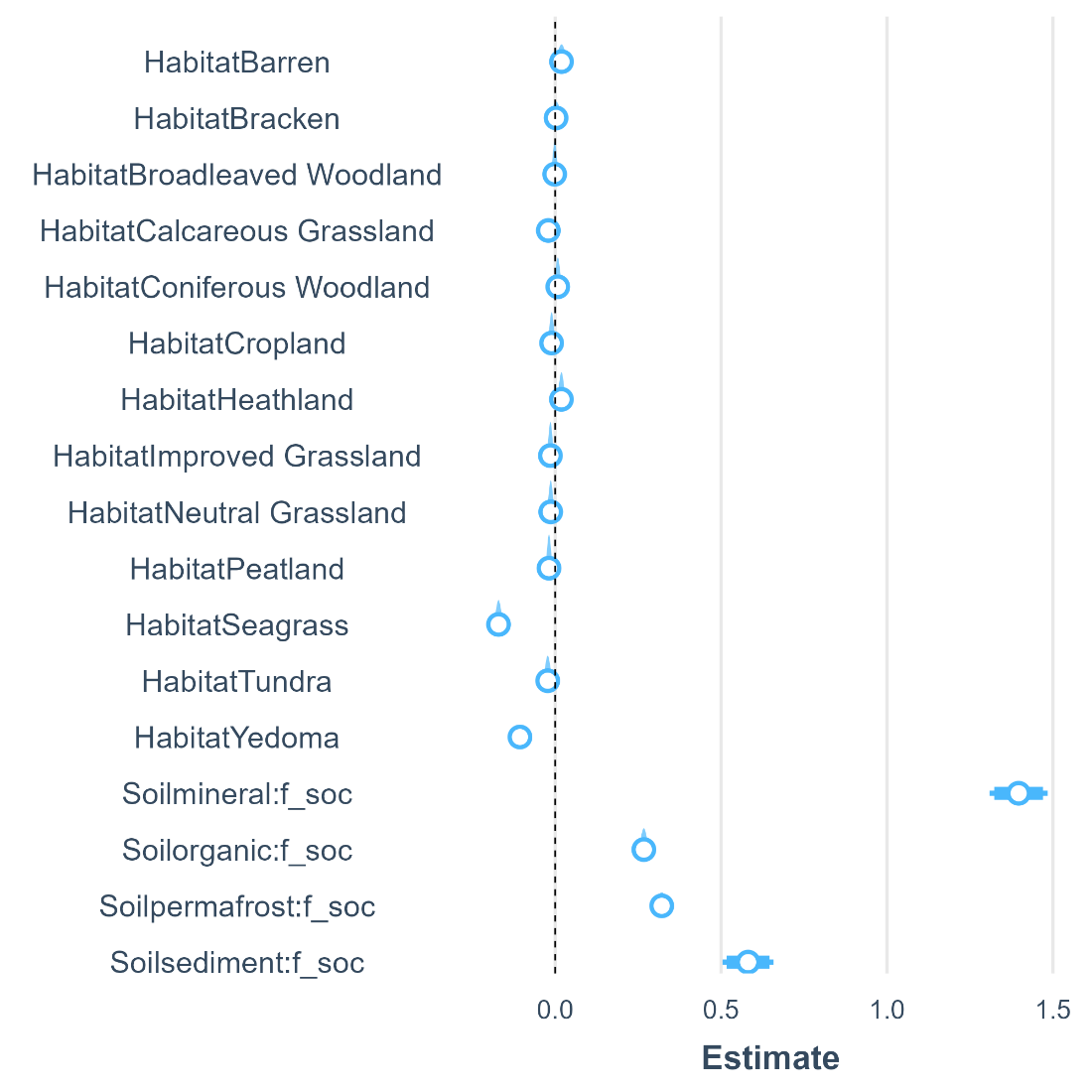


**Supplementary Fig. 15** SOC model output with model summary presented in Supplementary Table 4. Output for TC+SOC model is identical and not replicated here.

**Supplementary Table 1:** Overview of datasets used in the analysis of the main text: countries and their sampling extents, references to the datasets where available, information on loss on ignition (LOI) methods and references where available, number of data points included in the analysis, the average organic carbon content in soil organic matter ($f_{OC}$), or total carbon in SOM (f_oc_ or tc) where SOC was not available with associated *R^2^*. Information on data availability for SOM, total carbon (TC), and calcium carbonate contents (CaCO_3_). Datasets from the United Kingdom contain data from Scotland, England, and Wales. *Data from Croatia are not included in this comparison because SOC could not be calculated subtracting CaCO_3_ from TC (Supplementary Fig. 10); $ Data from the global saltmarsh database (Supplementary Fig. 9) are not included in our analysis but is analysed in Maxwell et al. (2023). The data used in this study can be found in Reinsch, Weber, et al. (2025).

| **Country** | **Extent** | **Reference** | **LOI temp (^o^C)** | **Amount of soil for LOI** | **Time** | **Method citations for LOI and EA** | **Number of data points** | **Max soil depth (cm)** | **Slope**  $f_{OC or tc}$ | ***R^2^*** | **SOM** | **TC** | **CaCO_3_** |
| --- | --- | --- | --- | --- | --- | --- | --- | --- | --- | --- | --- | --- | --- |
| United Kingdom | National | UKCEH Countryside Survey cycle 2020 (Bentley, Brentegani, et al., 2024; Bentley, Reinsch, Alison, Andrews, et al., 2023; Bentley, Reinsch, Alison, Brentegani, et al., 2023; Bentley, Reinsch, Brentegani, et al., 2023; Reinsch, Bentley, et al., 2023) | 105-375 | 10 g | 16 h | (Lebron et al., 2024) | 2134 | 15 | 0.5474 | 0.9953 | yes | yes | yes |
|  |  | UKCEH Countryside Survey 2021 winter survey (Bentley, Reinsch, et al., 2024) |  |  |  |  | 564 |  | 0.5274 | 0.9931 |  |  |  |
| Wales | National | (Reinsch, Bentley, et al., 2025; Wood et al., 2021) | 105-375 | 10 g | 16 h | (Lebron et al., 2024) | 352 | 15 | 0.5481 | 0.994 | yes | yes | yes |
| England | Targeted - habitat | (Reinsch, 2025) (Biosoil) | 105-375 | 10 g | 16 h | (Cools & De Vos, 2020) | 832 | 80 | 0.5482 | 0.9936 | Yes | Yes, and SOC | yes |
| Sweden | Targeted - region | (Palmtag, Obu, Kuhry, Richter, et al., 2022) | 105-550 | subsample of a 10 g sample | 5 h | (Palmtag et al., 2015), (Heiri et al., 2001) | 150 | 275 | 0.5001 | 0.9812 | yes | no, but SOC | yes |
| Norway |  |  |  |  |  |  | 123 |  |  |  |  |  |  |
| Russia |  |  |  |  |  |  | 804 |  |  |  |  |  |  |
| Canada |  |  |  |  |  |  | 84 |  |  |  |  |  |  |
| Greenland |  |  |  |  |  |  | 249 |  |  |  |  |  |  |
| Spain | Targeted - invited | (Reinsch, 2025) (Spain) | 105-375 | 10 g | 16 h | (Lebron et al., 2024) | 52 | 20 | 0.5237 | 0.9699 | yes | no, but SOC | no |
| Croatia * | Targeted - invited | Romic et al. (unpublished data) | 105-375 | 10 g | 16 h | (Lebron et al., 2024) | NA | 100 | NA | NA | yes | yes | yes |
| Finland | Targeted - habitat | (Heikkinen et al., 2021) | 105-550 | 2 g | Until constant weight (~6 h) | Leco TGA-710, ISO 11465 | 128 | 41 | 0.5035 | 0.9909 | yes | yes | no |
| United Kingdom | Targeted - habitat | (Toberman et al., 2016) | unknown-550 | unknown | 2 h | None mentioned | 307 | 417 | 0.5882 | 0.9968 | yes | yes | no |
| England | Targeted - function | (Reinsch et al., 2024; Reinsch, Lebron, et al., 2023; Smart et al., 2023) | 105-375 | 10 g | 16 h | (Lebron et al., 2024) | 368 | 100 | 0.5692 | 0.9961 | yes | yes | yes |
| United Kingdom | Targeted - habitat | (Reinsch, 2025) (CINAg) | 105-375 | 10 g | 16 h | None mentioned | 159 | 15 | 0.5522 | 0.9868 | yes | yes | no |
| United Kingdom | Targeted - habitat | (Mitchell et al., 2020) | 450 | unknown | unknown | None mentioned | 234 | 10 | 0.5261 | 0.9953 | yes | yes | no |
| Greenland | Targeted - habitat | Subset of samples in (Weber et al., 2021); Samples in (Weber et al., 2023) | 105-550 | 6 g | 12 h | (Hoogsteen et al., 2015) | 372 | 5 (directly below the turf layer) | 0.4858 | 0.9899 | yes | yes | yes |
| Switzerland | Targeted - habitat | (Leifeld et al., 2020) | NA | NA | NA | None mentioned | 1155 | 199 | 0.5386 | 0.9957 | yes | no, but SOC | no |
| Global $ | Targeted – habitat | (Maxwell et al., 2023) | Various | Various | Various | Various as reviewed in (Maxwell et al., 2023) | 1441 | 200 | 0.4068 | 0.9334 | Yes | no, but SOC | no |
| Global | Targeted – habitat | (Fourqurean, 2012; Fourqurean et al., 2012) | unknown-500/550 | Various | Various | Various as compiled in (Fourqurean et al., 2012) | 1384 | 475 | 0.436 | 0.9747 | yes | yes | No, but inorganic C |

**Supplementary Table 2** Summary of data by Habitat. N = Number of samples in each category (N=9503, the median, mean and standard deviation (SD) of the fraction of organic carbon in soil organic matter ($f_{OC}$), SOC = soil organic carbon (%), SOM = soil organic matter (%), mean = mean value (%), med = median (%), range = max – min (%) values for SOC and SOM, respectively. $f_{OC}$ median values relate to the order of habitats in Fig. 3.

| **Habitat** | **N** | $\boldsymbol{f}_{\boldsymbol{OC}}$ **med** | $\boldsymbol{f}_{\boldsymbol{OC}}$ **mean** | $\boldsymbol{f}_{\boldsymbol{OC}}$ **SD** | **SOC_mean_** | **SOC_med_** | **SOC_range_** | **SOM_mean_** | **SOM_med_** | **SOM_range_** |
| --- | --- | --- | --- | --- | --- | --- | --- | --- | --- | --- |
| Yedoma | 143 | 0.351 | 0.364 | 0.078 | 4.74 | 1.89 | 43.21 | 11.19 | 5.48 | 80.82 |
| Seagrass | 1384 | 0.367 | 0.360 | 0.089 | 2.96 | 2.17 | 48.23 | 7.74 | 5.93 | 99.69 |
| Tundra | 597 | 0.437 | 0.451 | 0.125 | 11.35 | 4.65 | 53.23 | 23.48 | 11.01 | 98.22 |
| Barren | 131 | 0.440 | 0.475 | 0.146 | 8.05 | 3.26 | 44.64 | 16.14 | 8.65 | 88.96 |
| Cropland | 610 | 0.460 | 0.469 | 0.083 | 2.59 | 2.21 | 40.95 | 5.44 | 4.82 | 70.55 |
| Neutral Grassland | 522 | 0.480 | 0.487 | 0.084 | 5.82 | 4.28 | 50.71 | 11.34 | 8.87 | 88.95 |
| Improved Grassland | 1024 | 0.485 | 0.493 | 0.063 | 5.93 | 4.72 | 46.96 | 11.84 | 9.64 | 84.40 |
| Broadleaved Woodland | 1259 | 0.507 | 0.497 | 0.087 | 6.65 | 3.61 | 54.48 | 12.71 | 7.22 | 94.68 |
| Calcareous Grassland | 17 | 0.518 | 0.505 | 0.033 | 7.61 | 6.96 | 17.98 | 14.85 | 13.72 | 33.23 |
| Bracken | 54 | 0.520 | 0.529 | 0.062 | 12.28 | 7.26 | 51.37 | 22.56 | 13.71 | 90.12 |
| Acid Grassland | 254 | 0.536 | 0.534 | 0.057 | 22.38 | 15.57 | 56.63 | 41.01 | 29.68 | 92.83 |
| Peatland | 2334 | 0.539 | 0.531 | 0.064 | 39.68 | 47.15 | 66.37 | 73.09 | 89.36 | 101.56 |
| Coniferous Woodland | 350 | 0.543 | 0.532 | 0.079 | 20.38 | 12.28 | 61.53 | 37.03 | 21.71 | 94.44 |
| Heathland | 257 | 0.557 | 0.556 | 0.072 | 29.60 | 27.81 | 59.04 | 53.41 | 51.55 | 93.15 |

**Supplementary Table 3** Summary of soil organic matter (SOM) fractionation data across habitats for 92 samples of the Countryside survey (Lebron et al., 2025). N = number of samples fractionated into particulate organic matter (POM) and mineral-associated organic matter (MAOM) per habitat and subsequently analysed for their soil organic carbon (SOC) contents. $f_{OC}$ = fraction of SOC in SOM, $f_{POC}$ = fraction of particulate organic carbon in SOC,
$f_{MAOC}$ = fraction of mineral associated organic carbon in SOC, SD, SE and CI are standard deviations, standard errors and 95% confidence intervals respectively. Data are plotted in Fig. 6, and the representative of $f_{OC}$ of this data subset is compared to the full dataset in Supplementary Fig. 4.

| **Habitat** | **N** | $\boldsymbol{f}_{\boldsymbol{OC}}$ | **SD** | **SE** | **CI** | $\boldsymbol{f}_{\boldsymbol{POC}}$ | **SD** | **SE** | **CI** | $\boldsymbol{f}_{\boldsymbol{MAOC}}$ | **SD** | **SE** | **CI** |
| --- | --- | --- | --- | --- | --- | --- | --- | --- | --- | --- | --- | --- | --- |
| Coniferous woodland | 2 | 0.467 | 0.005 | 0.003 | 0.041 | 0.545 | 0.019 | 0.014 | 0.175 | 0.455 | 0.019 | 0.014 | 0.175 |
| Cropland | 33 | 0.474 | 0.059 | 0.010 | 0.021 | 0.382 | 0.102 | 0.018 | 0.036 | 0.618 | 0.102 | 0.018 | 0.036 |
| Improved grassland | 19 | 0.485 | 0.046 | 0.010 | 0.022 | 0.408 | 0.138 | 0.032 | 0.067 | 0.592 | 0.138 | 0.032 | 0.067 |
| Neutral grassland | 19 | 0.500 | 0.058 | 0.013 | 0.028 | 0.419 | 0.124 | 0.028 | 0.060 | 0.581 | 0.124 | 0.028 | 0.060 |
| Calcareous grassland | 5 | 0.501 | 0.032 | 0.014 | 0.039 | 0.661 | 0.121 | 0.054 | 0.150 | 0.339 | 0.121 | 0.054 | 0.150 |
| Acid grassland | 4 | 0.535 | 0.017 | 0.008 | 0.027 | 0.515 | 0.092 | 0.046 | 0.147 | 0.485 | 0.092 | 0.046 | 0.147 |
| Broadleaved woodland | 8 | 0.538 | 0.088 | 0.031 | 0.074 | 0.666 | 0.182 | 0.064 | 0.152 | 0.336 | 0.180 | 0.064 | 0.151 |
| Heathland | 1 | 0.578 | NA | NA | NA | 0.988 | NA | NA | NA | 0.012 | NA | NA | NA |
| Bracken | 1 | 0.639 | NA | NA | NA | 0.721 | NA | NA | NA | 0.279 | NA | NA | NA |

**Supplementary Table 4** Statistical outputs for the applied *lmer* model in R using either the complete dataset (soil organic carbon (SOC) and total carbon (TC) where SOC was not available) as shown in Fig. 2 = TC+SOC model or removing data points where only TC was available (SOC model). The intercept is that of Acid Grassland to which the other habitats are compared to.

| **Habitat** | **TC model** | **TC model 95% CI** | **SOC model** | **SOC model 95% CI** |
| --- | --- | --- | --- | --- |
| (Intercept) | 0.54*** | 0.53, 0.55 | 0.54*** | 0.53, 0.55 |
| Barren | -0.03** | -0.05, -0.01 | -0.02* | -0.04, -0.00 |
| Bracken | 0.02 | -0.00, 0.04 | 0.02 | -0.00, 0.04 |
| Broadleaved woodland | 0.01 | -0.00, 0.02 | 0.00 | -0.01, 0.01 |
| Calcareous grassland | -0.01 | -0.04, 0.01 | 0.01 | -0.03, 0.04 |
| Coniferous woodland | 0.01 | -0.00, 0.02 | 0.01 | -0.01, 0.02 |
| Cropland | -0.01 | -0.02, 0.00 | -0.01 | -0.02, 0.00 |
| Heathland | 0.01 | -0.01, 0.02 | 0.01 | -0.01, 0.02 |
| Improved grassland | 0.01 | -0.01, 0.02 | -0.00 | -0.01, 0.01 |
| Neutral grassland | -0.00 | -0.01, 0.01 | -0.00 | -0.01, 0.01 |
| Peatland | -0.03*** | -0.04, -0.02 | -0.03*** | -0.04, -0.02 |
| Seagrass | -0.18*** | -0.20, -0.16 | -0.18*** | -0.19, -0.16 |
| Tundra | -0.07*** | -0.08, -0.06 | -0.07*** | -0.08, -0.05 |
| Yedoma | -0.11*** | -0.15, -0.08 | -0.11*** | -0.14, -0.08 |
| mineral:SOC | 0.10*** | 0.09, 0.11 | 0.09*** | 0.08, 0.10 |
| organic:SOC | 0.02*** | 0.01, 0.02 | 0.02*** | 0.02, 0.02 |
| permafrost:SOC | 0.07*** | 0.06, 0.07 | 0.07*** | 0.06, 0.07 |
| sediment:SOC | 0.11*** | 0.09, 0.12 | 0.11*** | 0.10, 0.13 |
| N |  | 9503 |  | 8936 |
| N (Profile) |  | 4710 |  | 4143 |
| AIC |  | -23332.42 |  | -21991.87 |
| BIC |  | -23189.23 |  | -21849.91 |
| R2 (fixed) |  | 0.57 |  | 0.57 |
| R2 (total) |  | 0.74 |  | 0.74 |

**Supplementary Table 5** The predicated fraction of carbon in soil organic matter, predicted $f_{OC}$ values, extracted from the SOC model with the results shown in the Supplementary Table S4. Results are plotted in Fig. 4 in the main text. Standard error (Std error), lower and higher confidence intervals (Conf low and high) for each Habitat and Soil category the habitats occur on. N is the number of data points for each Habitat x Soil category combination. Mineral and organic category equates to less than and more than 20% SOM, respectively. Sediments are found in Seagrass habitats and Yedoma sediments. Permafrost is assigned to data from the Permafrost region other than Yedoma sediments.

| **Habitat** | **Predicted** $\boldsymbol{f}_{\boldsymbol{OC}}$ | **Std error** | **Conf low** | **Conf high** | **Soil category** | **N** |
| --- | --- | --- | --- | --- | --- | --- |
| Acid Grassland | 0.494 | 0.005 | 0.484 | 0.504 | mineral | 105 |
| Bracken | 0.496 | 0.010 | 0.476 | 0.516 | mineral | 40 |
| Broadleaved Woodland | 0.491 | 0.003 | 0.486 | 0.497 | mineral | 1216 |
| Calcareous Grassland | 0.472 | 0.018 | 0.437 | 0.507 | mineral | 30 |
| Coniferous Woodland | 0.490 | 0.004 | 0.482 | 0.498 | mineral | 162 |
| Cropland | 0.483 | 0.003 | 0.477 | 0.489 | mineral | 752 |
| Heathland | 0.513 | 0.005 | 0.503 | 0.524 | mineral | 46 |
| Improved Grassland | 0.478 | 0.002 | 0.474 | 0.483 | mineral | 995 |
| Neutral Grassland | 0.479 | 0.003 | 0.473 | 0.485 | mineral | 525 |
| Peatland | 0.478 | 0.004 | 0.471 | 0.485 | mineral | 84 |
| Acid Grassland | 0.566 | 0.005 | 0.556 | 0.576 | organic | 154 |
| Bracken | 0.568 | 0.011 | 0.548 | 0.589 | organic | 14 |
| Broadleaved Woodland | 0.564 | 0.004 | 0.555 | 0.572 | organic | 164 |
| Calcareous Grassland | 0.544 | 0.018 | 0.509 | 0.580 | organic | 2 |
| Coniferous Woodland | 0.562 | 0.004 | 0.555 | 0.570 | organic | 783 |
| Cropland | 0.556 | 0.004 | 0.547 | 0.564 | organic | 154 |
| Heathland | 0.586 | 0.005 | 0.576 | 0.595 | organic | 211 |
| Improved Grassland | 0.551 | 0.004 | 0.543 | 0.559 | organic | 94 |
| Neutral Grassland | 0.551 | 0.004 | 0.543 | 0.559 | organic | 357 |
| Peatland | 0.550 | 0.003 | 0.544 | 0.556 | organic | 821 |
| Barren | 0.481 | 0.009 | 0.464 | 0.499 | permafrost | 131 |
| Broadleaved Woodland | 0.460 | 0.003 | 0.454 | 0.467 | permafrost | 129 |
| Coniferous Woodland | 0.459 | 0.004 | 0.451 | 0.468 | permafrost | 9 |
| Peatland | 0.447 | 0.004 | 0.439 | 0.455 | permafrost | 401 |
| Tundra | 0.440 | 0.005 | 0.430 | 0.450 | permafrost | 597 |
| Seagrass | 0.284 | 0.005 | 0.274 | 0.294 | sediment | 1384 |
| Yedoma | 0.349 | 0.015 | 0.319 | 0.378 | sediment | 143 |

References

Ahmad, M., & Subawi, H. (2013). New Van Krevelen diagram and its correlation with the heating value of biomass. *Journal of Agriculture and Environmental Management*, *2*(10), 295–301.

Bentley, L., Brentegani, M., Andrews, C., Brown, K., Cope, S., Ebuele, V., Emmett, B., Fitos, E., Friend, R., Garbutt, R., Gray, A., Hancock, G., Henrys, P., Higgins, A., Keenana, P., Keith, A., Lebron, I., Moscrop, A., O’Brien, L., … Robinson, D. (2024). Topsoil physico-chemical properties from the UKCEH Countryside Survey, Great Britain, 2023. *NERC EDS Environmental Information Data Centre*. https://doi.org/https://doi.org/10.5285/fe58d52e-f00c-4895-a35e-26b7353cc275

Bentley, L., Reinsch, S., Alison, J., Andrews, C., Brentegani, M., Chetiu, N., Dart, S., Dhiedt, E., Emmett, B. A., Fitos, E., Garbutt, R. A., Gray, A., Henrys, P. A., Hunt, A., Keenan, P. O., Keith, A. M., Koblizek, E., Lebron, I., Millani Lopes Mazzetto, J., … Robinson, D. A. (2023). Topsoil physico-chemical properties from the UKCEH Countryside Survey, Great Britain, 2018-2019. *NERC EDS Environmental Information Data Centre*. https://doi.org/https://doi.org/10.5285/821325f3-b353-4a51-8db2-6b2200d82aca

Bentley, L., Reinsch, S., Alison, J., Brentegani, M., Chetiu, N., Dhiedt, E., Emmett, B. A., Fitos, E., Garbutt, R. A., Guyatt, H., Henrys, P. A., Hunt, A., Keenan, P. O., Keith, A. M., Lebron, I., Long, C., Meek, B., Millani Lopes Mazzetto, J., O’Rourke, C., … Robinson, D. A. (2023). Topsoil physico-chemical properties from the UKCEH Countryside Survey, Great Britain, 2020, v2. *NERC EDS Environmental Information Data Centre*. https://doi.org/https://doi.org/10.5285/6a3382cc-f5f4-4d68-9517-c62fadd8af4f

Bentley, L., Reinsch, S., Brentegani, M., Chetiu, N., Dhiedt, E., Emmett, B., Fitos, E., Garbutt, R., Guyatt, H., Henrys, P., Hunt, A., Jackson-Bue, M., Keenan, P., Keith, A., Lebron, I., Pallett, D., Pereira, M., Risser, H., Rowe, R., … Robinson, D. (2023). Topsoil physico-chemical properties from the UKCEH Countryside Survey, Great Britain, 2021. *NERC EDS Environmental Information Data Centre*. https://doi.org/https://doi.org/10.5285/af6c4679-99aa-4352-9f63-af3bd7bc87a4

Bentley, L., Reinsch, S., Dhiedt, E., Henrys, P., Emmett, B., Risser, H., Tandy, S., Lebron, I., Brentegani, M., Garbutt, A., Wood, C., & Robinson, D. (2024). Winter topsoil physico-chemical properties from the UKCEH Countryside Survey, Great Britain, 2021. *NERC EDS Environmental Information Data Centre*. https://doi.org/https://doi.org/10.5285/38d6f5b4-4d03-4006-aa55-91894378ef27

Cools, N., & De Vos, B. (2020). *Part X: Sampling and Analysis of Soil. Version 2020-1. In: UNECE ICP Forests Programme Co-ordinating Centre (ed.): Manual on methods and criteria for harmonized sampling, assessment, monitoring and analysis of the effects of air pollution on forests*. https://www.icp-forests.org/pdf/manual/2020/ICP_Manual_part10_2020_Soil_version_2020-1.pdf

Emmett, B. A., Reynolds, B., Chamberlain, P. M., Rowe, E., Spurgeon, D., Brittain, S. A., Frogbrook, Z., Hughes, S., Lawlor, A. J., Poskitt, J., Potter, E., Robinson, D. A., Scott, A., Wood, C., & Woods, C. (2010). *Countryside Survey: Soils Report from 2007*. http://nora.nerc.ac.uk/9354/1/CS_UK_2007_TR9.pdf

Even, R. J., Machmuller, M. B., Lavallee, J. M., Zelikova, T. J., & Cotrufo, M. F. (2024). Large errors in common soil carbon measurements due to sample processing. *EGUsphere*, *2024*, 1–28. https://doi.org/10.5194/egusphere-2024-1470

Fernandez, C. W., & Kennedy, P. G. (2018). Melanization of mycorrhizal fungal necromass structures microbial decomposer communities. *Journal of Ecology*, *106*(2), 468–479. https://doi.org/https://doi.org/10.1111/1365-2745.12920

Fourqurean, J. (2012). *Global seagrass soil Corg data [Dataset]*. Florida Coastal Everglades LTER Program. https://fcelter.fiu.edu/data/other-data-resources/

Fourqurean, J. W., Duarte, C. M., Kennedy, H., Marbà, N., Holmer, M., Mateo, M. A., Apostolaki, E. T., Kendrick, G. A., Krause-Jensen, D., McGlathery, K. J., & Serrano, O. (2012). Seagrass ecosystems as a globally significant carbon stock. *Nature Geoscience*, *5*(7), 505–509. https://doi.org/10.1038/ngeo1477

Heikkinen, J., Keskinen, R., Kostensalo, J., & Nuutinen, V. (2022). Climate change induces carbon loss of arable mineral soils in boreal conditions. *Global Change Biology*, *28*(12), 3960–3973. https://doi.org/https://doi.org/10.1111/gcb.16164

Heikkinen, J., Keskinen, R., Regina, K., Honkanen, H., & Nuutinen, V. (2021). Estimation of carbon stocks in boreal cropland soils - methodological considerations. *European Journal of Soil Science*, *72*(2), 934–945. https://doi.org/https://doi.org/10.1111/ejss.13033

Heiri, O., Lotter, A. F., & Lemcke, G. (2001). Loss on ignition as a method for estimating organic and carbonate content in sediments: reproducibility and comparability of results. *Journal of Paleolimnology*, *25*(1), 101–110. https://doi.org/10.1023/A:1008119611481

Heldal, M., Norland, S., & Tumyr, O. (1985). X-ray microanalytic method for measurement of dry matter and elemental content of individual bacteria. *Applied and Environmental Microbiology*, *50*(5), 1251–1257. https://doi.org/10.1128/aem.50.5.1251-1257.1985

Hockaday, W. C., Purcell, J. M., Marshall, A. G., Baldock, J. A., & Hatcher, P. G. (2009). Electrospray and photoionization mass spectrometry for the characterization of organic matter in natural waters: a qualitative assessment. *Limnology and Oceanography: Methods*, *7*(1), 81–95. https://doi.org/https://doi.org/10.4319/lom.2009.7.81

Holmer, M., Duarte, C. M., Boschker, H. T. S., & BarrÃÂ^3^n, C. (2004). Carbon cycling and bacterial carbon sources in pristine and impacted Mediterranean seagrass  sediments. *Aquatic Microbial Ecology*, *36*(3), 227–237. https://www.int-res.com/abstracts/ame/v36/n3/p227-237/

Hoogsteen, M. J. J., Lantinga, E. A., Bakker, E. J., Groot, J. C. J., & Tittonell, P. A. (2015). Estimating soil organic carbon through loss on ignition: effects of ignition conditions and structural water loss. *European Journal of Soil Science*, *66*(2), 320–328. https://doi.org/https://doi.org/10.1111/ejss.12224

Jiang, Z., Zhao, C., Yu, S., Liu, S., Cui, L., Wu, Y., Fang, Y., & Huang, X. (2019). Contrasting root length, nutrient content and carbon sequestration of seagrass growing in offshore carbonate and onshore terrigenous sediments in the South China Sea. *Science of The Total Environment*, *662*, 151–159. https://doi.org/https://doi.org/10.1016/j.scitotenv.2019.01.175

Keith, A. M., Griffiths, R. I., Henrys, P. A., Hughes, S., Lebron, I., Maskell, L. C., Ogle, S. M., Robinson, D. A., Rowe, E. C., Smart, S. M., Spurgeon, D., Wood, C. M., & Emmett, B. A. (2020). Monitoring Soil Natural Capital and Ecosystem Services by Using Large-Scale Survey Data. In *Soil Ecosystems Services* (pp. 127–155). John Wiley & Sons, Ltd. https://doi.org/https://doi.org/10.2136/2015.soilecosystemsservices.2014.0070

Kuwata, M., Zorn, S. R., & Martin, S. T. (2012). Using Elemental Ratios to Predict the Density of Organic Material Composed of Carbon, Hydrogen, and Oxygen. *Environmental Science & Technology*, *46*(2), 787–794. https://doi.org/10.1021/es202525q

Lebron, I., Cooper, D. M., Brentegani, M. A., Bentley, L. A., Dos Santos Pereira, G., Keenan, P., Cosby, J. B., Emmet, B., & Robinson, D. A. (2024). Soil carbon determination for long-term monitoring revisited using thermo-gravimetric analysis. *Vadose Zone Journal*, *23*(1), e20300. https://doi.org/https://doi.org/10.1002/vzj2.20300

Lebron, I., Reinsch, S., Bentley, L., Cosby, B., Emmett, B., Keenan, P., Pereira, G., & Robinson, D. (2025). *Carbon and nitrogen contents of soil organic matter fractions, soil organic carbon, total soil nitrogen, and soil organic matter of 100 topsoil samples from the UKCEH Countryside Survey, Great Britain, 2019-2020 v2*. NERC EDS Environmental Information Data Centre. https://doi.org/10.5285/29cd5386-bc2e-4d70-a6a5-0d7dc7513dc6

Leifeld, J., Klein, K., & Wüst-Galley, C. (2020). Soil organic matter stoichiometry as indicator for peatland degradation. *Scientific Reports*, *10*(1), 7634. https://doi.org/10.1038/s41598-020-64275-y

Lu, Q., Tian, S., & Wei, L. (2023). Digital mapping of soil pH and carbonates at the European scale using environmental variables and machine learning. *Science of The Total Environment*, *856*, 159171. https://doi.org/https://doi.org/10.1016/j.scitotenv.2022.159171

Luo, H., Liu, S., Trevathan-Tackett, S. M., Ren, Y., Liang, J., Li, J., Jiang, Z., Wu, Y., & Huang, X. (2024). Nitrogen enrichment decreases seagrass contributions to refractory organic matter pools. *Limnology and Oceanography*, *69*(2), 367–379. https://doi.org/https://doi.org/10.1002/lno.12490

Ma, S., He, F., Tian, D., Zou, D., Yan, Z., Yang, Y., Zhou, T., Huang, K., Shen, H., & Fang, J. (2018). Variations and determinants of carbon content in plants: a global synthesis. *Biogeosciences*, *15*(3), 693–702. https://doi.org/10.5194/bg-15-693-2018

Maxwell, T. L., Rovai, A. S., Adame, M. F., Adams, J. B., Álvarez-Rogel, J., Austin, W. E. N., Beasy, K., Boscutti, F., Böttcher, M. E., Bouma, T. J., Bulmer, R. H., Burden, A., Burke, S. A., Camacho, S., Chaudhary, D. R., Chmura, G. L., Copertino, M., Cott, G. M., Craft, C., … Worthington, T. A. (2023). Global dataset of soil organic carbon in tidal marshes. *Scientific Data*, *10*(1), 797. https://doi.org/10.1038/s41597-023-02633-x

Mitchell, R. J., Hewison, R. L., Beaton, J., & Douglass, J. R. (2021). Identifying substitute host tree species for epiphytes: The relative importance of tree size and species, bark and site characteristics. *Applied Vegetation Science*, *24*(2), e12569. https://doi.org/https://doi.org/10.1111/avsc.12569

Mitchell, R. J., Hewison, R. L., Beaton, J., Haghi, R. K., Robertson, A. H. J., Main, A. M., Owen, I. J., & Douglass, J. (2020). Functional and epiphytic biodiversity differences between nine tree species in the UK. *NERC Environmental Information Data Centre*. https://doi.org/https://doi.org/10.5285/f539567f-a8cd-482e-89b8-64a951b52d93

Palmtag, J., Hugelius, G., Lashchinskiy, N., Tamstorf, M. P., Richter, A., Elberling, B., & Kuhry, P. (2015). Storage, landscape distribution, and burial history of soil organic matter in contrasting areas of continuous permafrost. *Arctic, Antarctic, and Alpine Research*, *47*(1), 71–88.

Palmtag, J., Obu, J., Kuhry, P., Richter, A., Siewert, M. B., Weiss, N., Westermann, S., & Hugelius, G. (2022). A high spatial resolution soil carbon and nitrogen dataset for the northern permafrost region based on circumpolar land cover upscaling. *Earth System Science Data*, *14*(9), 4095–4110. https://doi.org/10.5194/essd-14-4095-2022

Palmtag, J., Obu, J., Kuhry, P., Siewert, M., Weiss, N., & Hugelius, G. (2022). *Detailed pedon data on soil carbon and nitrogen for the northern permafrost region. Dataset version 1.* Bonin Centre Database. https://doi.org/https://doi.org/10.17043/palmtag-2022-pedon-1

Panagos, P., Montanarella, L., Barbero, M., Schneegans, A., Aguglia, L., & Jones, A. (2022). Soil priorities in the European Union. *Geoderma Regional*, *29*, e00510. https://doi.org/https://doi.org/10.1016/j.geodrs.2022.e00510

Pella, E., & Colombo, B. (1973). Study of carbon, hydrogen and nitrogen determination by combustion-gas chromatography. *Microchimica Acta*, *61*(5), 697–719. https://doi.org/10.1007/BF01218130

Pribyl, D. W. (2010). A critical review of the conventional SOC to SOM conversion factor. *Geoderma*, *156*(3), 75–83. https://doi.org/https://doi.org/10.1016/j.geoderma.2010.02.003

Reinsch, S. (2025). *Soil organic carbon and soil organic matter measurements across habitats from the UK and Spain (Version 1)*. Zenodo. https://doi.org/https://doi.org/10.5281/zenodo.17064291

Reinsch, S., Bentley, L., Brentegani, M., Carter, H., Dhiedt, E., Ebuele, V., Emmett, B., Hunt, A., Keenan, P., Ketih, A., Lebron, I., Lucas, J., Monkman, G., Pereira, M., Richardson-Jones, V., Rowe, R., Salisbury, E., Tandy, S., Wood, C., … Robinson, D. (2025). Topsoil physico-chemical properties from the Environment and Rural Affairs Monitoring & Modelling Programme, Wales, 2021-2023. *NERC EDS Environmental Information Data Centre*. https://doi.org/https://doi.org/10.5285/deabe608-fc6e-4d3a-812f-fb08ae515121

Reinsch, S., Bentley, L., Brentegani, M., Chetiu, N., Dhiedt, E., Emmett, B. A., Fitos, E., Garbutt, R. A. ; G. H., Henrys, P. A., Hunt, A., Keenan, P., Keith, A. M., Lebron, I., Mensah, E., Pallett, D. W., Pereira, M. G., Risser, H., Rowe, R. L., Salisbury, E., … Robinson, D. A. (2023). Topsoil physico-chemical properties from the UKCEH Countryside Survey, Great Britain, 2022. *NERC EDS Environmental Information Data Centre*. https://doi.org/https://doi.org/10.5285/d53fdf1d-767a-4046-821a-ea645001ddd3

Reinsch, S., Lebron, I., Brentegani, M., Brooks, M., Busi, S. B., Cagnarini, C., Cooper, D., Day, J., Emmett, B. A., Fitos, E., Goodall, T., Griffiths, R., Jones, B., Keenan, P., Keith, A., Lopes-Mazzetto, J. M., Mason, K. E., Pallett, D., Pereira, M. G., … Cosby, B. J. (2024). Co-located ecological data for exploring top- and subsoil carbon dynamics across grassland-woodland contrasts. *Scientific Data*, *11*(1), 478. https://doi.org/10.1038/s41597-024-03333-w

Reinsch, S., Lebron, I., Brentegani, M., Brooks, M., Busi, S., Cagnarini, C., Cooper, D., Day, J., Emmett, B., Fitos, E., Goodall, T., Griffiths, R., Jones, B., Keenan, P., Keith, A., Lopes-Mazzetto, J., Mason, K., Pallett, D., Pereira, G., … Cosby, B. (2023). Soil physical, chemical, and biological properties (0-1 m) at five long-term grassland-to-woodland land use contrasts across England, 2018-2019. In *NERC EDS Environmental Information Data Centre*. https://doi.org/https://doi.org/10.5285/8a2451f8-7999-47c4-9e2a-8e9567377ffb

Reinsch, S., Weber, P. L., Vanguelova, E., Bentley, L., Heikkinen, J., Leifeld, J., Hugelius, G., & Robinson, D. A. (2025). *Soil organic carbon and soil organic matter measurements across European habitats, including permafrost regions and global seagrass sediments (Version 1) [Data set]*. Zenodo. https://doi.org/10.5281/zenodo.17305831

Reynolds, B., Chamberlain, P. M., Poskitt, J., Woods, C., Scott, W. A., Rowe, E. C., Robinson, D. A., Frogbrook, Z. L., Keith, A. M., Henrys, P. A., Black, H. I. J., & Emmett, B. A. (2013). Countryside Survey: National “Soil Change” 1978–2007 for Topsoils in Great Britain—Acidity, Carbon, and Total Nitrogen Status. *Vadose Zone Journal*, *12*(2), vzj2012.0114. https://doi.org/https://doi.org/10.2136/vzj2012.0114

Rich, C. I., & Kunze, G. W. (1964). *Soil clay mineralogy (a symposium). North Carolina: Uni*. Carolina Press.

Robinson, D. A., Bentley, L., Jones, L., Feeney, C., Garbutt, A., Tandy, S., Lebron, I., Thomas, A., Reinsch, S., Norton, L., Maskell, L., Wood, C., Henrys, P., Jarvis, S., Smart, S., Keith, A., Seaton, F., Skates, J., Higgins, S., … Emmett, B. A. (2024). Five decades’ experience of long-term soil monitoring, and key design principles, to assist the EU soil health mission. *European Journal of Soil Science*, *75*(5), e13570. https://doi.org/https://doi.org/10.1111/ejss.13570

Smart, S., Cagnarini, C., Cosby, B., Emmett, B., Thomas, A., & Reinsch, S. (2023). Plant aboveground net primary productivity estimates (2021) and litter layer depth measurements (2018-2019) at five long-term grassland-to-woodland land use contrasts across England. In *NERC EDS Environmental Information Data Centre*. https://doi.org/https://doi.org/10.5285/9156d263-4def-406d-bdc9-22a6821fe755

Soinne, H., Kurkilahti, M., Heikkinen, J., Eurola, M., Uusitalo, R., Nuutinen, V., & Keskinen, R. (2022). Decadal trends in soil and grain microelement concentrations indicate mainly favourable development in Finland. *Journal of Plant Nutrition and Soil Science*, *185*(5), 578–588. https://doi.org/https://doi.org/10.1002/jpln.202200141

Toberman, H., Tipping, E., Somerville, C., Helliwell, R., Carter, H., Keenan, P., Dos Santos Pereira, G., Patel, M., Tanna, B., Thompson, N., Bryant, C., Elliott, F., & Gulliver, P. (2016). *Peat survey in England, Scotland and Wales carried out during 2014 [LTLS]*. https://doi.org/10.5285/9305b068-f417-4659-9966-d9456f22c331

Van Krevelen, D. W. (1950). Graphical-statistical method for the study of structure and reaction processes of coal. *Fuel*, *29*, 269–284.

Vanguelova, E. I., Nisbet, T. R., Moffat, A. J., Broadmeadow, S., Sanders, T. G. M., & Morison, J. I. L. (2013). A new evaluation of carbon stocks in British forest soils. *Soil Use and Management*, *29*(2), 169–181. https://doi.org/https://doi.org/10.1111/sum.12025

Weber, P. L., Blaesbjerg, N. H., Moldrup, P., Pesch, C., Hermansen, C., Greve, M. H., Arthur, E., & de Jonge, L. W. (2023). Organic carbon controls water retention and plant available water in cultivated soils from South Greenland. *Soil Science Society of America Journal*, *87*(2), 203–215. https://doi.org/https://doi.org/10.1002/saj2.20490

Weber, P. L., Hermansen, C., Nørgaard, T., Pesch, C., Moldrup, P., Greve, M. H., Arthur, E., & de Jonge, L. (2022). Evaluating the particle densities of subarctic soils using pedotransfer functions and vis–NIR spectroscopy. *Soil Science Society of America Journal*, *86*(4), 964–978. https://doi.org/https://doi.org/10.1002/saj2.20410

Weber, P. L., Hermansen, C., Norgaard, T., Pesch, C., Moldrup, P., Greve, M. H., Müller, K., Arthur, E., & de Jonge, L. W. (2021). Moisture-dependent Water Repellency of Greenlandic Cultivated Soils. *Geoderma*, *402*, 115189. https://doi.org/https://doi.org/10.1016/j.geoderma.2021.115189

Wood, C. M., Alison, J., Botham, M. S., Burden, A., Edwards, F., Garbutt, R. A., George, P. B. L., Henrys, P. A., Hobson, R., Jarvis, S., Keenan, P., Keith, A. M., Lebron, I., Maskell, L. C., Norton, L. R., Robinson, D. A., Seaton, F. M., Scarlett, P., Siriwardena, G. M., … Emmett, B. A. (2021). Integrated ecological monitoring in Wales: the Glastir Monitoring and Evaluation Programme field survey. *Earth System Science Data*, *13*(8), 4155–4173. https://doi.org/10.5194/essd-13-4155-2021

Zamanian, K., Taghizadeh-Mehrjardi, R., Tao, J., Fan, L., Raza, S., Guggenberger, G., & Kuzyakov, Y. (2024). Acidification of European croplands by nitrogen fertilization: Consequences for carbonate losses, and soil health. *Science of The Total Environment*, *924*, 171631. https://doi.org/https://doi.org/10.1016/j.scitotenv.2024.171631

Zhang, J., & Elser, J. J. (2017). Carbon: nitrogen: phosphorus stoichiometry in fungi: a meta-analysis. *Frontiers in Microbiology*, *8*, 1281.
